# Methyl 4‐*O*‐Demethylbarbatate Destabilises HIF‐1α and Enhances Temozolomide Sensitivity in Glioma Cells

**DOI:** 10.1111/cpr.70279

**Published:** 2026-09-27

**Authors:** Rui Zhou, Mücahit Varlı, Rundong Liu, Jieun Yu, Hyung‐Ho Ha, Jae‐Seoun Hur, Sang Kyum Kim, Hahyun Park, Hangun Kim

**Affiliations:** ^1^ College of Pharmacy, Sunchon National University Sunchon Jeollanam‐do Republic of Korea; ^2^ Department of Microbiology, Tumor and Cell Biology Karolinska Institute Stockholm Sweden; ^3^ Korea Lichen Research Institute Sunchon National University Sunchon Republic of Korea; ^4^ Ningbo Institute of Marine Medicine Peking University Ningbo China; ^5^ College of Pharmacy, Chungnam National University Daejeon Republic of Korea

**Keywords:** energy metabolism, epithelial‐mesenchymal transition, glioma, HIF‐1α, lichen‐derived natural products, stemness

## Abstract

Gliomas remain among the most lethal brain tumours, driven by aggressive invasion, stem‐like properties, metabolic plasticity, and frequent resistance to temozolomide (TMZ). Hypoxia‐inducible factor‐1α (HIF‐1α) is a central regulator of these malignant traits. In this study, we identify methyl 4‐*O*‐demethylbarbatate (Me‐4‐*O*‐DBA), a biosynthetically produced lichen‐derived metabolite, as a potential modulator of HIF‐1α signaling. Molecular docking analysis suggested a possible interaction between Me‐4‐*O*‐DBA and HIF‐1α near the Lys304 ubiquitination site, which may contribute to reduced HIF‐1α stability and suppression of downstream EMT and stemness signaling. This inhibition simultaneously attenuated both glycolytic activity and mitochondrial respiration, indicating a broad suppression of metabolic reprogramming. Network pharmacology and molecular docking further indicated HIF‐1α‐associated signaling as a potential regulatory hub, while in vitro assays demonstrated marked reductions in glioma cell viability, invasion, migration, and clonogenicity. Notably, Me‐4‐*O*‐DBA synergised with TMZ to enhance suppression of motility, stemness, and metabolic activity, resulting in profound impairment of tumourigenic potential. In vivo zebrafish assays also indicated a favourable safety profile at the tested concentrations. Together, these findings suggest that Me‐4‐*O*‐DBA suppresses HIF‐1α‐associated malignant phenotypes and enhances TMZ responsiveness in glioma cells, highlighting its potential as a natural product‐derived modulator of glioma progression.

## Graphical abstract

The lichen‐derived metabolite methyl 4‐O‐demethylbarbatate (Me‐4‐O‐DBA) suppresses glioma stemness, motility, and metabolic plasticity through inhibition of HIF‐1α signaling and synergistically enhances temozolomide activity, highlighting a potential strategy to target stemness‐driven therapeutic resistance in glioma.

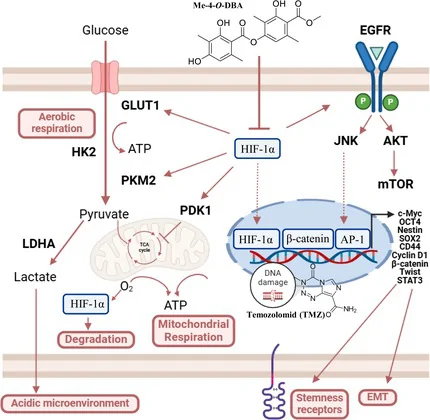

**Abbreviations**
ATP: adenosine triphosphate
CHD4: chromodomain helicase DNA binding protein 4
CoCl_2_
: cobalt (II) chloride
DMSO: dimethyl sulfoxide
DNA: deoxyribonucleic acid
ECAR: extracellular acidification rate
EMT: epithelial‐mesenchymal transition
FCCP: carbonyl cyanide 4‐(trifluoromethoxy) phenylhydrazone
GLUT1: glucose transporter 1
HIF‐1α: hypoxia‐inducible factor‐1 alpha
HK2: hexokinase 2
LDHA: L‐actate dehydrogenase A
mRNA: messenger ribonucleic acid
MTT: 3‐(4,5‐dimethyl‐2‐thiazolyl)‐3,5‐diphenyl‐2H‐tetrazolium bromide
NADH: nicotinamide adenine dinucleotide
OCR: oxygen consumption rate
PDK1: pyruvate dehydrogenase kinase 1
PGC‐1α: peroxisome proliferator‐activated receptor gamma coactivator 1‐alpha
PKM2: pyruvate kinase M2
RNA: ribonucleic Acid
SAM: S‐adenosyl‐L‐methionine
SRC‐1: steroid receptor coactivator‐1
TME: tumour microenvironment
TMZ: temozolomide

## 1 Introduction

Gliomas, the most common malignant primary brain tumours, are characterised by local invasiveness, complex oncogenic mechanisms, and poor overall survival rates [1]. Current treatment strategies, primarily radiation and chemotherapy, focus on inducing DNA damage [2]. However, mutations in oncogenes and tumour suppressors drive rapid cancer progression and increase resistance to these therapies [3, 4]. Temozolomide (TMZ), marketed as Temodar, is a widely used DNA‐alkylating agent in glioblastoma treatment, demonstrating effectiveness in both primary and recurrent cases and significantly improving patient survival. However, despite its clinical success, tumour recurrence and resistance remain major challenges [5, 6]. Therefore, novel therapeutic strategies are urgently needed to improve glioma patient outcomes.

Epithelial‐mesenchymal transition (EMT), stemness, and metabolism are interconnected processes that play crucial roles in both physiological and pathological conditions, particularly in cancer progression. EMT, defined by the transition of epithelial cells into mesenchymal‐like cells, enhances migratory and invasive properties, facilitating metastasis [7]. Stemness, the ability of cells to self‐renew and differentiate, is closely linked to EMT and contributes to tumour heterogeneity and therapy resistance [8, 9]. Additionally, metabolic reprogramming, a hallmark of cancer, supports the energetic and biosynthetic demands of rapidly proliferating tumour cells, promoting growth and survival. Emerging evidence highlights the intricate crosstalk among EMT, stemness, and metabolism, with each process regulating the others to drive cancer progression and therapeutic resistance [10]. A deeper understanding of these interconnections could provide novel therapeutic insights for combating cancer and improving patient outcomes.

The tumour microenvironment (TME) has become a major focus of cancer research, as it plays a critical role in tumour growth and progression. Beyond the complex interactions between cancer cells and neighbouring non‐neoplastic cells, hypoxia, driven by the rapid proliferation of cancer cells, is a key factor shaping the TME in most solid tumours [11]. Reduced oxygen activity causes the alpha subunits of hypoxia‐inducible factors to accumulate and hinder their breakdown [12]. HIF‐1α, in particular, is a major regulator of cancer metabolism, immune evasion, angiogenesis, EMT, and cell motility, all of which contribute to tumour aggressiveness [13, 14]. Consequently, targeting HIF‐1α, its co‐transcriptional factors, and its downstream signaling pathways presents a promising therapeutic strategy for enhancing cancer treatment efficacy [15].

Natural compounds have long been a major source of anticancer drugs due to their unique mechanisms of action. Extensive research has revealed the chemical diversity and biological potential of lichen‐derived natural products, particularly their effects against various cancer types [16]. Notably, lichen secondary metabolites, including atranorin, have demonstrated anticancer activity in hepatocellular carcinoma, breast cancer, melanoma, lung cancer, and glioblastoma [17, 18, 19, 20, 21, 22]. In our previous study, we linked a gene cluster to atranorin, the major secondary metabolite of lichens, by genetic duplication and heterologous expression. We discovered several compounds on our way to reconstructing the atranorin biosynthetic pathway [23]. One of these strains produced 4‐*O*‐demethylbarbatic acid, which exhibited therapeutic effects against EGFR‐driven lung cancer [24]. Another strain produced methyl 4‐*O*‐demethylbarbatate (Me‐4‐*O*‐DBA), though its anticancer mechanism remains unclear. Given the therapeutic potential of depside‐containing natural products, including atranorin and its derivatives, further investigation into their anticancer properties is warranted [25]. In this study, we evaluate the mechanism of action of Me‐4‐*O*‐DBA in glioma, highlighting its potential for sustainable industrial production via biosynthesis. Furthermore, our findings demonstrate that the combined treatment with Me‐4‐*O*‐DBA and TMZ modulates key processes in glioma, including oncogenic signaling, stemness, EMT, and cellular metabolism.

## 2 Materials and Methods

### 2.1 Materials

The strain utilised was the genetically modified *Ascochyta rabiei* strain, as described in the previous paper [23]. Co‐expression of two biosynthetic genes in this engineered strain, *atr1* and *atr3*, yielded methyl 4‐*O*‐demethylbarbatate (Me‐4‐*O*‐DBA), which was annotated as an oxidised aldehyde of atranorin. TMZ was obtained from Sigma‐Aldrich (T2577).

### 2.2 Extraction and Purification

To conduct biological evaluation, we purified Me‐4‐*O*‐DBA through a large‐scale culture of this strain expressing *atr1* and *atr3*. The strain was first cultivated on culture dishes of potato‐dextrose agar at 25°C for 10 days. Then the agar was cut into grain size to inoculate in flasks. Hundreds of 500‐mL Fernbach, each containing 80 g of rice and 120 mL of distilled water, were autoclaved at 120°C for 30 min, in which the fermentation proceeded. After cooling to room temperature, each of the flasks was inoculated with 10 mL of the spore inoculum and cultured at 25°C for 30 days. Subsequently, the ferment culture was extracted with ethyl acetate (three times, each sonication for 45 min) and vacuum‐dried to afford the crude extract. Crude extracts were reconstituted with methanol and subsequently purified using 95% methanol (*t*_*R*_ = 9.60 min; 200 mg) using high‐performance liquid chromatography (HPLC).

Methyl 4‐*O*‐demethylbarbatate (Me‐4‐*O*‐DBA): white solid; ^1^H‐NMR (600 MHz, DMSO‐D6) *δ* 11.15 (s, 1H), 10.52 (s, 1H), 10.33 (s, 1H), 6.69 (s, 1H), 6.38 (s, 1H), 3.87 (s, 3H), 2.48 (s, 3H), 2.33 (s, 3H), 1.96 (d, J = 2.6 Hz, 6H), ^1^H‐NMR (600 MHz, DMSO‐D6, D_2_O) *δ* 6.60 (s, 1H), 6.35 (s, 1H), 3.82 (s, 3H), 2.43 (s, 3H), 2.29 (s, 3H), 1.91 (d, *J* = 4.5 Hz, 6H). MS (ESI‐, m/z): 359 [M‐H] (Figures S1–S3).

### 2.3 Feeding Experiment of *A. rabiei* Strain Expressing *atr3*

*Ascochyta rabiei* ‘clean host’ strains carrying either the gene atr3 or an empty vector were cultured in 100 mL of the corresponding PDB medium at 20°C with shaking at 150 rpm for 6 days. Subsequently, the substrate 4‐*O*‐demethylbarbatic acid was added individually to the cultures at a final concentration of 10 μg/mL. The cultures were incubated for an additional 4 days under the same conditions. After incubation, the cultures were extracted with ethyl acetate, followed by sonication and evaporation of the organic solvent. The resulting extracts were dissolved in 200 μL of methanol, and 10 μL was subjected to HPLC analysis.

### 2.4 ADMET Information Collection of Me‐4‐*O*‐DBA and Atranorin

pkCSM (http://biosig.unimelb.edu.au/pkcsm/prediction) databases and PubChem (https://pubchem.ncbi.nlm.nih.gov/) were used to collect the ADMET information.

### 2.5 Cell Culture and Reagents

The human glioma cell lines U87 and U251 were purchased from the American Type Culture Collection (ATCC; Manassas, VA, USA), while the mouse glioma cell line GL261 was generously provided by Dr. Maciej S. Lesniak (University of Chicago). Cancer cells were cultured in DMEM/RPMI1640 (Gendepot) supplemented with 10% fetal bovine serum (GIBCO) and 1% penicillin–streptomycin solution in a humidified atmosphere containing 5% CO_2_ at 37°C. MG132 (Proteasome inhibitor; Sigma‐Aldrich), Baf A1 (Bafilomycin A1 from *Streptomyces griseus* , autophagy inhibitor; Sigma‐Aldrich), CQ (Chloroquine, autophagy inhibitor; Sigma‐Aldrich), 3‐MA (3‐Methyladenine, autophagy inhibitor; Sigma‐Aldrich), TSA (trichostatin A, HDAC inhibitor; Selleckchem).

### 2.6 MTT Assay

2.5 × 10^4^ cells/mL of Caco‐2, MCF‐7, AGS, U87, U251, and GL261 cells were seeded in a 96‐well plate and incubated overnight and treated with DMSO, 8.7–278 μM of Me‐4‐*O*‐DBA and atranorin for 48 h. Then, cultures were supplemented with 15 μL of MTT reagent (Sigma‐Aldrich) for 4 h. The culture medium was removed and then incubated for 10 min with 150 μL DMSO at 37°C. The density values of cells were measured at 540 nm by a microplate reader and analysed by Gen5 (2.03). IC_50_ values were calculated using SPSS version 23.

### 2.7 Transwell Invasion Assay

Invasion assays were performed in 8 μm transwell chambers (Corning Inc., Corning) with 1% gelatin‐coated polycarbonate membranes. 2–2.5 × 10^6^ cells/mL of cells were seeded in DMEM/RPMI1640 medium containing 0.2% bovine serum albumin in the upper chambers and incubated overnight. A 10 μg/mL fibronectin was additionally added to the lower chambers. For single treatment, cells were treated with either 0.01% of DMSO and Me‐4‐*O*‐DBA (7, 14, and 28 μM). For combination treatment, cells were treated with either 0.01% of DMSO, 14 μM of Me‐4‐*O*‐DBA, 200 μM of TMZ, and 14 μM of Me‐4‐*O*‐DBA/TMZ (200 μM), respectively. After treatment for 24 h, cells in upper chambers were fixed using a Diff Quik Kit (Sysmex, Kobe). The stained cells were counted under a light microscope (five fields per chamber) and measured by iSolution FL Auto ×64 software (IMT i‐Solution Inc., Northampton).

### 2.8 Spheroid Formation Assay

About 2.5–5 × 10^3^ cells/mL of U87, U251 and GL261 cells were seeded into ultra‐low attachment 24‐well plates with the N2‐supplemented DMEM/F12 medium (Invitrogen) containing 10 ng/mL human recombinant epidermal growth factor (Biovision) and 10 ng/mL human basic fibroblast growth factor (Invitrogen). After incubation for 3 h, cells were treated with 0.01% DMSO and Me‐4‐*O*‐DBA (7, 14, and 28 μM) for single treatment. For combination treatment, cells were treated with either 0.01% DMSO, 14 μM of Me‐4‐*O*‐DBA, 200 μM of TMZ, and 14 μM of Me‐4‐*O*‐DBA + TMZ (200 μM), respectively. After incubation for 7–14 days, spheres were counted using a Nikon Eclipse 400 fluorescence microscope (Nikon Instech Co. Ltd.) and measured through iSolution FL Auto ×64 software (IMT i‐Solution Inc., Northampton).

### 2.9 Clonogenic Assay

0.5–1 × 10^3^ cells/mL of cells were seeded into 6‐well plates. After incubation for 3 h, cells were treated with 0.01% DMSO and Me‐4‐*O*‐DBA (7, 14, and 28 μM). Subsequently, cells were incubated for 10–14 days at 37°C. The colonies were stained with 0.05% of crystal violet staining solution. Pixel intensity of colony area was measured by iSolution FL Auto x64 software (IMT i‐Solution Inc., Northampton).

### 2.10 Cell Cycle Arrest Assay

U87 and U251cells (2 × 10^5^ cells/mL) were seeded into 6‐well plates and incubated for 12 h, and then treated with 0.01% of DMSO, Me‐4‐*O*‐DBA (14 and 28 μM), TMZ (200 μM), and TMZ plus Me‐4‐*O*‐DBA (14 and 28 μM) for 48 h. Cells were incubated with trypsin solution after harvesting and washing with FACS washing buffer. Cells were then incubated with ribonuclease A for 10 min at RT. Pellets were stained with 100 μL of 4 mg/mL PI (Sigma–Aldrich) for an additional 2 h in the dark at 4°C. The results were analysed by flow cytometer (Beckman Coulter Life Sciences) and CytoFLEX software version 2.0 (Beckman Coulter Life Sciences).

### 2.11 Cellular Thermal Shift Assay (CETSA)

U87 and U251 cells were seeded and allowed to attach overnight. Cells were then treated with Me‐4‐O‐DBA (14 μM) in the presence of cobalt (II) chloride (CoCl_2_, 100 μM) for 12 h to induce HIF‐1α accumulation. After treatment, cells were washed twice with ice‐cold PBS, detached with trypsin, and collected by centrifugation.

Cell suspensions were aliquoted into PCR tubes and subjected to heat treatment at 40°C, 45°C, 50°C, or 55°C for 3 min. Immediately after heating, samples were snap‐frozen in liquid nitrogen and thawed in a 37°C water bath. This freeze–thaw cycle was repeated three times to facilitate cell lysis. The lysates were then centrifuged at 20,000 × g for 20 min at 4°C to remove aggregated and denatured proteins. The resulting supernatants containing soluble proteins were collected and mixed with SDS loading buffer. Band intensities were quantified using Multi Gauge 3.0 software (Fujifilm, Tokyo, Japan), and HIF‐1α signals were normalised to α‐tubulin. Thermal stability curves were generated by plotting the normalised protein levels against temperature. The relative soluble HIF‐1α level at each temperature was expressed as a percentage of that detected at 40°C.

### 2.12 Cycloheximide Chase Assay

To evaluate the effect of Me‐4‐O‐DBA on HIF‐1α protein stability, U87 and U251 cells were seeded and cultured overnight. Cells were pretreated with CoCl_2_ (100 μM) for 6 h to induce HIF‐1α accumulation, followed by treatment with Me‐4‐O‐DBA (14 μM) for 12 h. Subsequently, cycloheximide (CHX; 50 μg/mL) was added to block de novo protein synthesis. Cells were harvested at the indicated time points (0, 20, 40, 60, and 80 min) after CHX treatment. Equal amounts of protein were separated by SDS–PAGE and transferred onto PVDF membranes. HIF‐1α protein levels were detected by Western blotting using specific antibodies, with α‐tubulin serving as a loading control. Protein band intensities were quantified using Multi Gauge 3.0 software (Fujifilm, Tokyo, Japan). HIF‐1α levels were normalised to α‐tubulin and expressed relative to the corresponding level at time 0. The degradation kinetics of HIF‐1α were analysed by plotting the normalised protein levels against time after CHX treatment.

### 2.13 Ubiquitination Assay

Briefly, U87 and U251 cells were pretreated with CoCl_2_ (100 μM) for 6 h and subsequently incubated with Me‐4‐O‐DBA for 6 h. Cells were lysed and clarified by centrifugation at 14,000 × g for 15 min at 4°C. Equal amounts of protein (500 μg) were incubated overnight at 4°C with anti‐HIF‐1α antibody, followed by incubation with Protein A/G agarose beads for 1 h at room temperature. The immune complexes were washed five times with ice‐cold wash buffer. Bound proteins were eluted by boiling in 1× LDS sample buffer containing reducing agent at 70°C for 10 min. Ubiquitinated HIF‐1α was detected by immunoblotting with anti‐ubiquitin antibody.

### 2.14 Combination Index (CI)

The combination effects or index (CI) of Me‐4‐*O*‐DBA and TMZ were calculated using CompuSyn software as described [26]. A CI < 1 means a synergistic effect, whereas CI equal to 1 or > 1 indicate additive and antagonist effects, respectively.

### 2.15 Scratch Wound Healing Assay

Cell migration assay was performed with an IncuCyte ZOOM HD system (Sartorius, Göttingen, Germany). About 2 × 10^4^ cells/well of U87 and U251were plated in Essen ImageLock 96‐well plates. After incubation overnight, the scratch wound was made on the monolayer of confluent cells using Wound Marker (Sartorius). After wounding, cell debris was cleared by PBS, and the cells were incubated in corresponding culture medium (DMEM) supplemented with 2% FBS with the Me‐4‐*O*‐DBA (14 μM), TMZ (200 μM), and Me‐4‐*O*‐DBA + TMZ. The progression of the wound healing in each well was obtained at 4 h intervals for a total of 24 h. The data were analysed using relative wound density (RWD) by the IncuCyte Software (Sartorius, Göttingen, Germany).

### 2.16 Quantitative Real‐Time PCR (qRT‐PCR)

Briefly, total RNA was extracted from glioma cells using RNAiso Plus (TaKaRa, Otsu, Japan). Total RNA (1 μg) from treated cells was converted to cDNA using a M‐MLV Reverse Transcriptase kit (Invitrogen). SYBR Green (Enzynomics) was used to determine relative gene expression. Quantitative real‐time PCR analysis was performed using CFX (Bio‐Rad, Hercules). The primers used for real‐time PCR are listed in Table S1.

### 2.17 Immunoblot Analysis

U87 and U251 cells were treated with 0.01% DMSO or the indicated concentrations of Me‐4‐*O*‐DBA and TMZ for 24 or 48 h; the expression of EMT, CSC, and cancer metabolism markers were then investigated. Protein samples were separated by SDS–PAGE and transferred to PVDF membranes following standard procedures. To ensure accurate normalisation and clear band resolution, either β‐actin or α‐tubulin was used as the loading control depending on the molecular weight of the target proteins in each experiment. Specifically, β‐actin (≈42 kDa) or α‐tubulin (≈53 kDa) was selected to avoid overlap with the proteins of interest and to prevent signal interference. This strategy ensured optimal separation between target proteins and loading controls, thereby allowing reliable quantification. All experiments were performed under identical electrophoresis and transfer conditions, and band intensities were quantified using Multi Gauge 3.0 software, and their relative densities were calculated against α‐tubulin or β‐actin band density. Antibody information was given in Table S2.

### 2.18 Seahorse Analysis of Extracellular Acidification Rate and Oxygen Consumption Rate Measurements

Briefly, U87 and U251 cells were seeded at 5–10 × 10^4^ cells/mL, incubated overnight in 2% FBS of DMEM medium, and then treated with 0.01% DMSO, 14 μM of Me‐4‐*O*‐DBA, 200 μM of TMZ, and 14 μM of Me‐4‐*O*‐DBA + TMZ (200 μM) for 24 h, respectively. The cells were washed twice and incubated (180 μL) with DMEM assay medium enriched with glucose, sodium pyruvate, and glutamine for 1 h at 37°C in a non‐CO_2_ incubator prior to analysis. About 0.5 μM rotenone/antimycin A and 50 mM 2‐deoxy‐d‐glucose (2‐DG) were loaded into the hydrated sensor cartridge for glycolytic rate analysis and 1 μM oligomycin, 1 μM carbonyl cyanide 4‐(trifluoromethoxy) phenylhydrazone (FCCP), and 0.5 μM rotenone (Rot) + antimycin A (AA) were loaded for Cell Mito Stress Test. glycoPER and oxygen consumption rate were measured on a Seahorse XF instrument (Agilent). OCR and ECAR values were normalised to the number of cells viability per well. The data were analysed using the Wave XFp Analyser software.

### 2.19 KEGG Pathway Enrichment

Firstly, the structure of Me‐4‐*O*‐DBA was imported into the SwissTarget Prediction and PharmMapper databases to obtain all the targets. Then, the related genes were obtained with the keyword ‘Glioma’ by GeneCards and Drug‐Bank databases. Finally, the Venny 2.1.0 database was used to find the candidate targets at the intersection. To investigate the biological pathways of pathogenic genes, Annotation, Visualisation, and Integrated Discovery (DAVID) and Cytoscape databases were used to analyse the candidate targets related to Kyoto Encyclopedia of Genes and Genomes (KEGG) pathways. Then, the top 15 KEGG pathways, with an adjusted *p* value of 0.05, were plotted by https://www.bioinformatics.com.cn, an online platform for data analysis and visualisation.

### 2.20 Screening of Hub Genes and Molecular Docking

A CTP network was constructed using Cytoscape 3.8.1 software to analyse the relationships between all identified targets. Subsequently, a PPI (protein–protein interaction) network was built using the STRING database to investigate the interactions among the 11 candidate genes of Me‐4‐*O*‐DBA and TMZ in glioma. The Cytohubba plug‐in was used to explore hub genes, and the top three were generated using maximal clique centrality (MCC), closeness and degree methods. Semi‐flexible molecular docking was used to study the binding energy and interaction sites between compounds and genes, as well as the position of amino acids. First, the PDB formats of the hub targets were downloaded from RSCBPDB, and the 3D structures of Me‐4‐*O*‐DBA and TMZ were downloaded from PubChem. After processing with PyMOL, they were imported into AutoDockTools‐1.5.6 for docking. By analysing the clusters in the results, the optimal conformations of Me‐4‐*O*‐DBA and TMZ and hub genes were obtained according to the binding energy (threshold is < −4 kcal/mol) and active binding sites. Finally, the maximum distance of hydrogen bond between compound and protein was set to 2 Å to ensure the accuracy of molecular docking. Then, the protein–ligand interaction profiler database was used to describe the binding amino acid residues of the compound and the protein in PDB database.

### 2.21 Acute Toxicity Test for Zebrafish Embryos

To evaluate the acute toxicity of Me‐4‐*O*‐DBA, zebrafish embryos were maintained in an embryo medium containing 0.03% 1‐phenyl‐2‐thiourea (PTU) to prevent pigmentation. Stock solutions of Me‐4‐*O*‐DBA and TMZ were prepared in DMSO and subsequently diluted in PTU medium to yield final concentrations of Me‐4‐*O*‐DBA at 3, 6, and 14 μM, and TMZ at 200 μM. A volume of 1 mL of each working solution was distributed into individual wells, with 24 embryos assigned to each concentration. The exposure media containing Me‐4‐*O*‐DBA and TMZ was refreshed every 24 h over the 72 h‐experimental period to minimise contamination. In accordance with OECD Test Guideline 236 for fish embryo acute toxicity, morphological assessments were conducted at 72 h using confocal microscopy, including observations of body length, yolk sac area, pericardial edema, and tail morphology. All experimental protocols were approved by the Institutional Animal Care and Use Committee (IACUC) of Sunchon National University (approval no. SCNU IACUC‐2024‐25).

### 2.22 Zebrafish Brain Tumour Models

Malignant glioma U87 cells labelled with fluorescent dye were collected and resuspended in fresh culture medium to achieve a final concentration of 2 × 10^8^ cells/mL, corresponding to approximately 200 cells per injection. Wild‐type zebrafish larvae (AB strain) at 48 h post‐fertilisation (hpf) were dechorionated and positioned in a 1% agarose mould to facilitate microinjection. The suspension of U87 cells mixed with fresh culture medium and matrigel was loaded into a capillary needle, then injected into the brain ventricle of anaesthetised larvae using a microinjector. While human glioma cells are typically maintained at 37°C, xenografted zebrafish larvae were incubated at 34°C, a temperature suitable for both zebrafish development and tumour cell viability [27]. At 24 h post‐injection, xenografted larvae were selected based on the fluorescence intensity of transplanted U87 cells visualised using the T‐red channel in confocal microscopy. The selected xenografted larvae were then treated with various concentrations of Me‐4‐*O*‐DBA and TMZ until the experimental endpoint at 120 hpf.

### 2.23 Statistical Analysis

All in vitro experiments were repeated at least three times. The student's *t*‐test and one‐way ANOVA analysis were used to determine significant differences. *p* < 0.05 was considered a statistically significant difference. Statistical analyses were carried out using IBM SPSS version 22 and Sigma Plot 12.5 software.

## 3 Results

### 3.1 Biosynthesis of Methyl 4‐*O*‐Demethylbarbatate

Previous studies have demonstrated that identifying the biosynthetic gene clusters (BGC) and expressing its genes in heterologous host can deepen our understanding of lichen metabolism and facilitate the discovery of novel bioactive compounds. In the atranorin biosynthetic pathway, 4‐*O*‐demethylbarbatic acid acts as a crucial intermediate and is converted into methyl 4‐*O*‐demethylbarbatate (Me‐4‐*O*‐DBA) by ATR3, an *O*‐methyltransferase encoded by the *atr3* gene, and subsequently converted into atranorin through the action of the *atr2* gene (Figure 1A). To validate the function of ATR3, we fed 4‐*O*‐demethylbarbatic acid to an *Ascochyta rabiei* ‘clean host’ strain overexpressing *atr3*. This resulted in the production of Me‐4‐*O*‐DBA, confirming that ATR3 functions as an *O*‐methyltransferase catalysing the methylation of the carboxylic acid group of 4‐*O*‐demethylbarbatic acid. In contrast, feeding the precursor to the *A. rabiei* ‘clean host’ strain carrying the empty vector had no effect, indicating that the conversion is dependent on *atr3* expression (Figure 1B). To investigate the enzymatic mechanism of ATR3, a structural model was generated using SWISS‐MODEL (Figure 1C). The Ramachandran plot analysis showed that 98.2% of residues are located in favoured or allowed regions (Figure 1D), indicating a reliable predicted structure. To further explore ATR3's interaction with its substrate and identify key residues involved in substrate recognition, molecular docking was performed between ATR3 and 4‐*O*‐demethylbarbatic acid, revealing multiple potential binding sites. Molecular docking analysis showed that three representative conformations of the ATR3–4‐*O*‐demethylbarbatic acid complex exhibited strong binding affinities, averaging −7.17 kcal/mol. The substrate interacted with ATR3 through hydrogen bonds involving residues Ala‐88, Lys‐81, and Arg‐156, specifically at the ester group, with bond distances ranging from 1.7 to 2.4 Å (Figure 1E). These results highlight the important role of hydrogen bonding in substrate binding and the formation of Me‐4‐*O*‐DBA.

**FIGURE 1:**
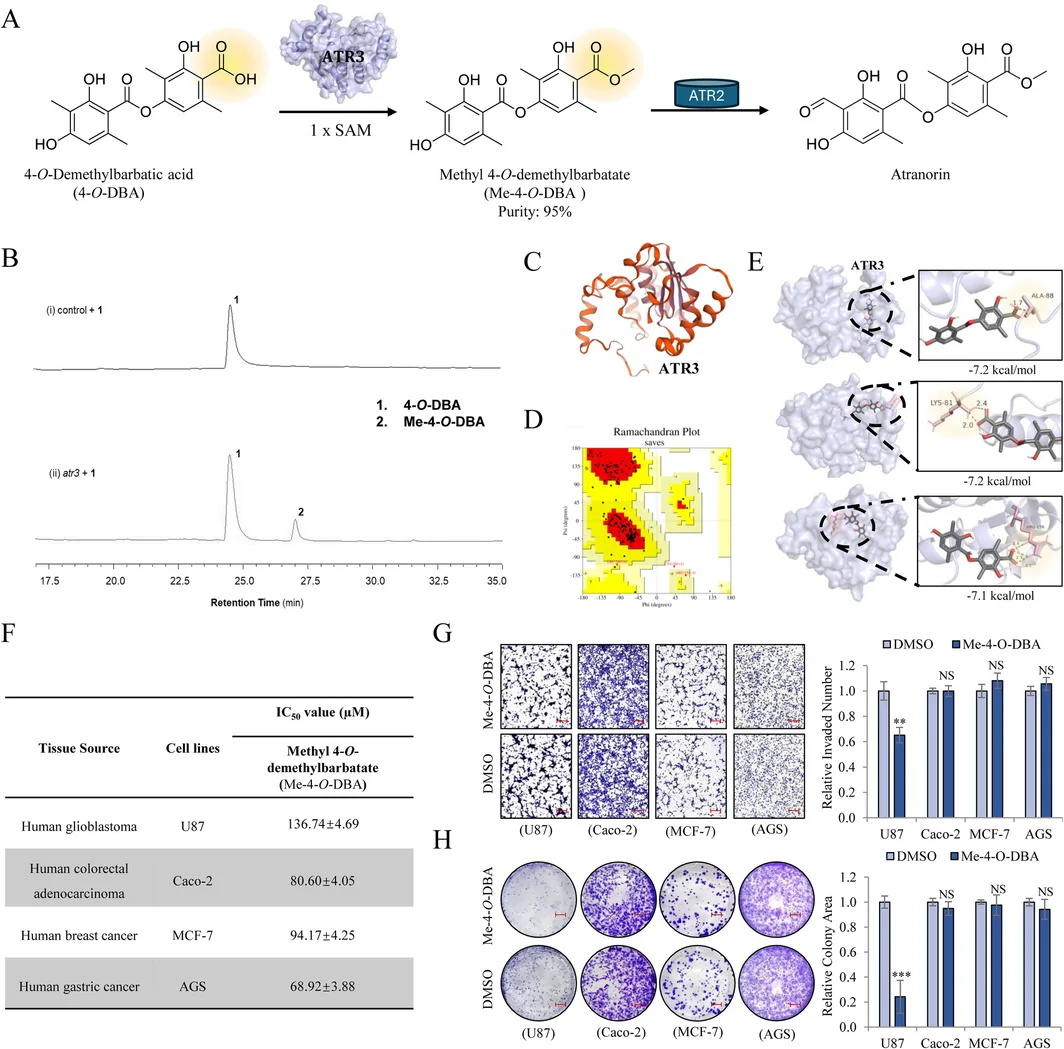
Biosynthesis of Me‐4‐*O*‐DBA and characterisation of ATR3 enzymatic function. (A) Proposed biosynthetic pathway for atranorin synthesis. The substrate 4‐*O*‐demethylbarbatic acid is methylated by ATR3, an *O*‐methyltransferase encoded by the atr3 gene, to produce Me‐4‐*O*‐DBA, which is subsequently converted into atranorin by ATR2. (B) HPLC chromatograms showing the conversion of 4‐*O*‐DBA into Me‐4‐*O*‐DBA. 4‐*O*‐DBA in control extract and formation of Me‐4‐*O*‐DBA upon feeding 4‐*O*‐DBA to *Ascochyta rabiei* overexpressing atr3, confirming ATR3's function as an *O*‐methyltransferase. Peaks: (1) 4‐*O*‐demethylbarbatic acid; (2) Me‐4‐*O*‐DBA. (C) Predicted structural model of ATR3 generated using SWISS‐MODEL. (D) Ramachandran plot analysis showing the structural reliability of the ATR3 model. (E) Representative docking conformations of the ATR3–4‐*O*‐demethylbarbatic acid complex, with yellow spheres indicating the interaction sites. (F) U87, Caco2, MCF‐7 and AGS cells treated with ranging concentrations (4.35–278 μM) of Me‐4‐*O*‐DBA for 48 h were assessed using the MTT assay. IC_50_ values were given for each cancer cell line. (G) Representative images of U87, Caco2, MCF‐7, and AGS, cells that invaded through transwell inserts coated with 1% gelatin after 24 h of exposure to a low concentration (28 μM) of Me‐4‐*O*‐DBA. Quantitative analysis of invaded cell counts is shown. Data are represented as the mean ± standard deviation (SD). **p < 0.01; NS indicates no significant difference compared to the DMSO‐treated group. (H) Representative images and quantitative results of clonogenic assays for different cancer cell lines treated with 28 μM of Me‐4‐*O*‐DBA for 7–14 days. Data are represented as the mean ± SD. ***p < 0.001; NS indicates no significant difference compared to the DMSO‐treated group.

### 3.2 Me‐4‐*O*‐DBA Inhibits Invasion, Spheroid Formation, and Proliferative Potential of Glioma Cells

Atranorin and its derivatives demonstrate potent inhibitory effects against various cancer types, including lung, breast, and brain cancers. To assess whether Me‐4‐*O*‐DBA possesses anti‐cancer activity, we measured its IC_50_ values on various cancer cell lines: U87 (136.74 μM), Caco‐2 (80.60 μM), MCF‐7 (94.17 μM), and AGS (68.92 μM) (Figure 1F). Notably, Me‐4‐*O*‐DBA selectively inhibited U87 cell invasion and colony formation by 35% and 76%, respectively (Figure 1G,H), highlighting its potent anti‐glioma activity. Furthermore, the viability of glioma cell lines U87, U251, and GL261 was assessed using an MTT assay. Treatment with 8.7–278 μM Me‐4‐*O*‐DBA for 48 h significantly reduced cell viability, with marked effects observed at concentrations above 69.5 μM (Figure 2A). The IC_50_ values of Me‐4‐*O*‐DBA were approximately 109–118 μM in all tested cells. Atranorin, used as a positive control, exhibited comparable IC_50_ values (107–119 μM) in U87, U251, and GL261 cells (Figure 2B). To evaluate the effects of Me‐4‐*O*‐DBA at noncytotoxic concentrations, additional experiments were performed using lower doses (7, 14, and 28 μM). Results revealed a dose‐dependent reduction in the number of invaded cells compared to the control (Figure 2C). Gliomas are known to contain self‐renewing, tumourigenic cancer stem cells (CSCs) that contribute to tumour progression and therapy resistance. Our data demonstrated that Me‐4‐*O*‐DBA significantly inhibited spheroid formation in a dose‐dependent manner (Figure 2D), suggesting suppression of CSC‐like properties. In addition, Me‐4‐*O*‐DBA suppressed the proliferative potential of glioma cells, as shown in Figure 2E. To elucidate the molecular mechanism underlying the anticancer activity of Me‐4‐*O*‐DBA, we investigated its impact on critical cellular pathways involved in motility and stemness. As shown in Figure 2F,H, treatment with Me‐4‐*O*‐DBA markedly reduced the protein levels of N‐cadherin, Snail, Slug, Twist (motility‐related markers), as well as Aldh1, Msi, Lgr5, and CD133 (stemness markers) in U87 cells. Consistently, the corresponding mRNA expression levels were also significantly decreased following treatment (Figure 2G,I). These results suggest that Me‐4‐*O*‐DBA broadly disrupts glioma cell phenotypes by downregulating both motility‐associated and stemness‐associated pathways.

**FIGURE 2:**
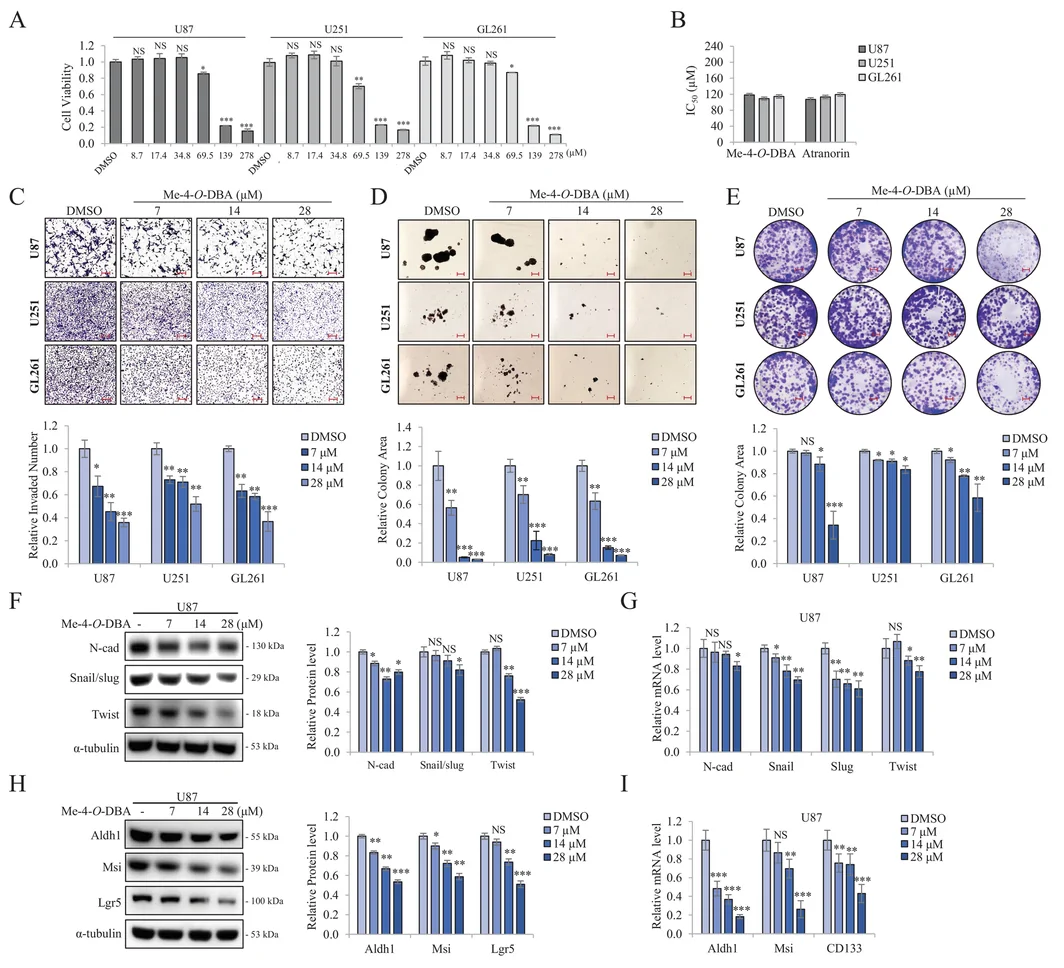
Effects of Me‐4‐*O*‐DBA on cell viability, invasion, spheroid formation, and proliferation in glioma cells. (A) Cell viability of U87, U251, and GL261 glioma cell lines treated with various concentrations of Me‐4‐*O*‐DBA. (B) IC_50_ values of Me‐4‐*O*‐DBA and atranorin in glioma cell lines. (C) Invasion assays of U87, U251, and GL261 cells treated with 7, 14, or 28 μM of Me‐4‐*O*‐DBA. Quantitative analysis of the number of invaded cells in each group. (D) Spheroid formation assays of glioma cells treated with 7, 14, or 28 μM of Me‐4‐*O*‐DBA for 7–14 days. Quantification of spheroid numbers following treatment. (E) Clonogenic assay of U87, U251, and GL261 cells treated with 7, 14, or 28 μM of Me‐4‐*O*‐DBA, with quantification of colony number in each group. Data represent the mean ± SD. *p < 0.05; **p < 0.01; ***p < 0.001; NS: No significant difference compared with DMSO‐treated cells. (F, H) Western blot analysis of the expression levels of proteins involved in EMT and stemness in the presence or absence of Me‐4‐*O*‐DBA in U87 cells. (G, I) Quantification of mRNA level of EMT markers (N‐cadherin, Slug, Snail, and Twist) and stemness markers (ALDH1, Msi, and CD133). Data represent the mean ± SD. *p < 0.05; **p < 0.01; 1; ***p < 0.001; NS: No significant difference compared with DMSO‐treated cells.

### 3.3 Me‐4‐*O*‐DBA Directly Interacts With HIF‐1α and Promotes Its Degradation in Glioma

Since HIF‐1α plays a crucial role in regulating the tumour hypoxic microenvironment and controlling the transcription and expression of downstream target genes, developing therapeutic drugs targeting HIF‐1α is of great significance. HIF‐1α is highly expressed in glioma cells, especially in U87 cells (Figure 3A). Using a gene expression profiling interactive analysis (GEPIA), we found that the expression levels of HIF‐1α were higher in patients with GBM (Glioblastoma multiforme) and LGG (Low grade glioma) than that in normal samples (Figure 3B). A total of 322 genes closely associated with brain cancer were obtained from GeneCards and Drug Bank, while 100 Me‐4‐*O*‐DBA‐targeted genes were obtained from SwissTarget Prediction and PharmMapper databases. A total of 22 overlapping genes were identified and analysed as candidate genes using the Venn 2.1.0 database (Figure 3C). KEGG enrichment analysis identified 15 significantly enriched signaling pathways (*p* < 0.01), including several cancer‐related pathways, with particular emphasis on the HIF‐1 signaling pathway. Our analysis confirmed that the identified target gene is closely associated with both glioma and HIF‐1α (Figure 3D). To further characterise the interaction between HIF‐1α and Me‐4‐*O*‐DBA, molecular docking results showed a binding affinity of −7.1 kcal/mol. Me‐4‐*O*‐DBA interacts with the hydrogen bonding region formed by Lys‐304 and Pro‐301 in the N‐terminal domain of HIF‐1α. Notably, Lys‐304 (K304) functions as a key ubiquitination site in the oxygen‐independent degradation of HIF‐1α, acting as a ubiquitin acceptor in a VHL‐independent pathway mediated by the RACK1/Elongin/Cullin E3 ligase complex [28, 29]. Based on this interaction, we propose that Me‐4‐*O*‐DBA facilitates the ubiquitination and degradation of HIF‐1α under hypoxic conditions by recruiting or stabilising an E3 ligase complex at the K304 region, with Pro‐301 contributing structurally to the recognition interface (Figure 3E). To determine whether HIF‐1α is a direct target of Me‐4‐*O*‐DBA, a cellular thermal shift assay was performed under CoCl_2_‐induced hypoxic conditions. As shown in Figure 3F, treatment with Me‐4‐*O*‐DBA significantly increased the thermal stability of HIF‐1α compared with the control group, indicating a direct interaction between Me‐4‐*O*‐DBA and HIF‐1α in glioma cells. We next examined whether Me‐4‐*O*‐DBA affected HIF‐1α protein stability using a cycloheximide (CHX) chase assay. Following inhibition by CHX, HIF‐1α protein levels decreased more rapidly in Me‐4‐*O*‐DBA‐treated cells than in control cells (Figure 3G), indicating that Me‐4‐*O*‐DBA shortened the half‐life of HIF‐1α and accelerated its degradation.

**FIGURE 3:**
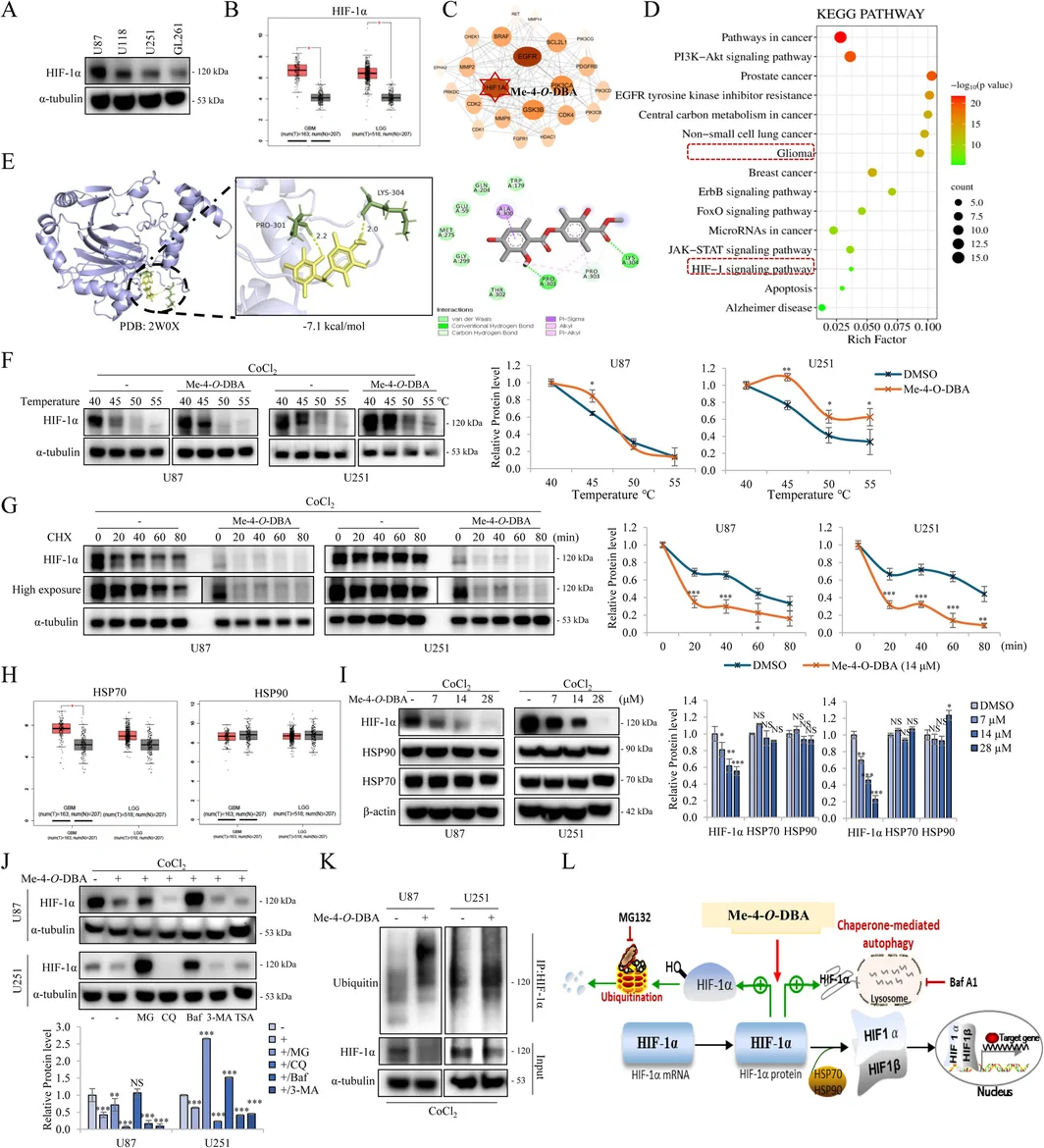
Me‐4‐*O*‐DBA directly binds to HIF‐1α and promotes its ubiquitination‐dependent trafficking and degradation. (A) The endogenous expression of HIF‐1α in U87, U118, U251, and GL261 cells. (B) HIF‐1α expression in GBM (*n* = 163) and LGG (*n* = 518) versus normal tissues (*n* = 207), analysed using GEPIA. (C) A PPI of overlapping therapeutic targets of Me‐4‐*O*‐DBA and brain cancer. (D) KEGG pathway enrichment analysis of target genes, highlighting glioma and HIF‐1 signaling pathways. (E) The predicted interactions of Me‐4‐*O*‐DBA with HIF‐1α (PDB ID: 2W0X). (F) CETSA showing thermal stabilisation of HIF‐1α upon Me‐4‐*O*‐DBA treatment in U87 and U251 cells. (G) U87 and U251 cells were treated with Me‐4‐*O*‐DBA followed by CHX (50 μg/mL), and HIF‐1α protein levels were analysed at the indicated time points by Western blotting. Quantification of HIF‐1α protein degradation kinetics following CHX treatment. (H) Box plots showing the expression levels of HSP70 and HSP90 in GBM and LGG from GEPIA database. The red and grey boxes represent tumour (T) and normal (N) tissue samples, respectively. (I) Western blot analysis of HSP70, HSP90, and HIF‐1α under hypoxia (CoCl_2_, 100 μM) after Me‐4‐*O*‐DBA treatment (7–28 μM). (J) Me‐4‐*O*‐DBA–induced HIF‐1α degradation. U87 and U251 cells were treated with 14 μM Me‐4‐*O*‐DBA for 12 h, followed by treatment with MG132 (10 μM), CQ (50 μM), Baf A1 (50 nM), 3‐MA (100 μM), or TSA (0.2 μM) for 16 h. Immunoreactive HIF‐1α was examined, with α‐tubulin as a loading control. Data represent the mean ± SD. *p < 0.05; **p < 0.01; 1; ***p < 0.001; NS: No significant difference compared with DMSO‐treated cells. (K) Ubiquitination assay showing increased ubiquitination of HIF‐1α after Me‐4‐*O*‐DBA treatment. (L) Schematic representation of the proposed mechanism of Me‐4‐*O*‐DBA action. Me‐4‐*O*‐DBA promotes HIF‐1α ubiquitination and subsequent degradation via chaperone‐mediated autophagy, leading to reduced HIF‐1α levels in glioma cells.

Heat shock protein family (HSP70/90) plays an important role in HIF‐1α stabilisation; we examined the effect of Me‐4‐*O*‐DBA on the expression of HSP70 and HSP90. The GEPIA database showed that HSP70 expression was upregulated in GBM and LGG compared to normal tissues, while HSP90 expression was not significantly changed (Figure 3H). Furthermore, under hypoxic conditions, Me‐4‐*O*‐DBA significantly inhibited the expression of HIF‐1α but had no effect on HSP70 and HSP90 (Figure 3I). To determine the degradation pathway of HIF‐1α downregulated by Me‐4‐*O*‐DBA, we evaluated the effects of several inhibitors. As shown in Figure 3J, treatment with the proteasome inhibitor MG132 and lysosomal inhibitor bafilomycin A1 (Baf A1) restored Me‐4‐*O*‐DBA‐mediated downregulation of HIF‐1α, suggesting that the proteasome and lysosomal degradation pathways were involved. However, treatment with autophagy inhibitor chloroquine (CQ) and a Class III PI3K inhibitor that blocks the early steps of autophagosome formation 3‐methyladenine (3‐MA) failed to restore Me‐4‐*O*‐DBA‐mediated HIF‐1α downregulation. While CQ primarily functions as a weak lysosomotropic base that elevates lysosomal pH and indirectly suppresses lysosomal protease activity, Baf strongly blocks lysosomal acidification and autophagosome‐lysosome fusion by directly inhibiting vacuolar type H+‐ATPase (V‐ATPase) [30, 31]. Our findings, showing that Baf, but not CQ, has the ability to restore HIF‐1α levels, suggest that Me‐4‐*O*‐DBA supports a fusion‐dependent lysosomal degradation pathway. Since histone deacetylases enhance the stability of HIF‐1α under hypoxic conditions and the transactivation function of HIF‐1α. Trichostatin A (TSA), A histone deacetylase inhibitor, was selected for detection. The results showed that TSA did not inhibit Me‐4‐*O*‐DBA‐induced HIF‐1α degradation. To investigate whether ubiquitination contributes to HIF‐1α degradation, ubiquitination assays were performed. Me‐4‐*O*‐DBA markedly increased the accumulation of ubiquitinated HIF‐1α species, indicating that Me‐4‐*O*‐DBA promotes HIF‐1α polyubiquitination (Figure 3K). These findings suggested that Me‐4‐*O*‐DBA‐mediated HIF‐1α downregulation is dependent on the ubiquitin‐proteasome pathway and chaperone‐mediated autophagy system in the lysosomes (Figure 3L). Taken together, these data demonstrated that Me‐4‐*O*‐DBA exerts anti‐glioma effects by targeting and reducing HIF‐1α expression.

### 3.4 Me‐4‐*O*‐DBA Synergises With TMZ to Inhibit Glioma Cell Motility and Spheroid Formation

The combination of Me‐4‐O‐DBA and TMZ significantly reduced glioma cell viability across most tested doses. Combination index (CI) analysis showed a synergistic effect (CI < 1) in most conditions, except in U87 cells treated with 7 μM Me‐4‐O‐DBA plus 100 μM TMZ (Figure 4A). In addition, flow cytometry analysis of PI‐stained U87 and U251 cells treated with Me‐4‐*O*‐DBA and TMZ showed no significant accumulation of cells in the SubG1 phase but an increased proportion in the G2/M phase at 200 μM TMZ and/or 14 μM Me‐4‐*O*‐DBA + 200 μM TMZ (Figure 4B). To assess the apoptotic effects of the combination treatment, Western blot analysis was performed to examine apoptosis‐related proteins, including PARP and caspase‐3. Notably, treatment with 200 μM TMZ or 14 μM Me‐4‐*O*‐DBA + 200 μM TMZ induced the expression of cleaved caspase‐3 (Figure 4C). Based on the CI values, the optimal combination for synergy was determined to be 14 μM Me‐4‐*O*‐DBA plus 200 μM TMZ (CI = 0.78 in U87; CI = 0.59 in U251), which was selected for further experiments. Moreover, these results confirm that Me‐4‐*O‐*DBA alone suppressed glioma cell motility, stemness, and proliferation independently of its cytotoxic effects at concentrations of 14–28 μM, highlighting the potent effect at a safe dose.

**FIGURE 4:**
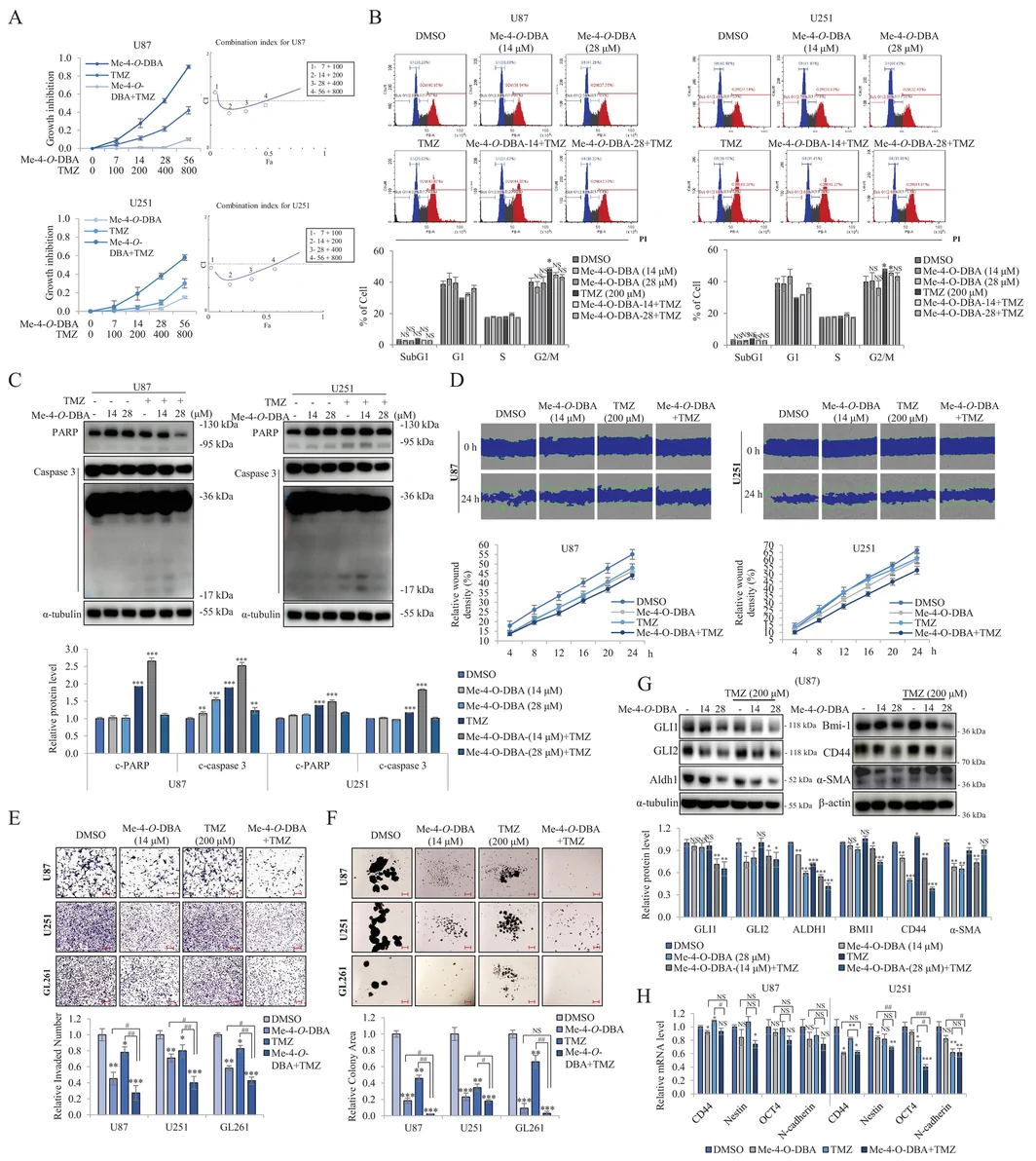
Combination effect of Me‐4‐*O*‐DBA and TMZ on glioma cell motility, spheroid formation, and the related gene expression. (A) Cell viability and CI analysis (CompuSyn) of U87 and U251 cells treated with Me‐4‐*O*‐DBA (7, 14, 28 or 56 μM) and TMZ (100, 200, 400, or 800 μM) alone or in combination for 48 h. (B) Cell cycle distribution of U87 and U251 cells treated with Me‐4‐*O*‐DBA (14, 28 μM), TMZ (200 μM), or their combination for 48 h, analysed by flow cytometry. (C) Western blot analysis of PARP and caspase‐3 following the same treatments, with α‐tubulin as loading control. (D) Wound healing assay showing migration of U87, U251, and GL261 cells after 24 h treatment with Me‐4‐*O*‐DBA (14 μM), TMZ (200 μM), or combination. Representative images of wound closure and quantitative analysis of relative wound density are shown. (E) Transwell invasion assay under the same treatment conditions. Representative images and quantification of invaded cell numbers are displayed. (F) Spheroid formation assay of glioma cells treated with Me‐4‐*O*‐DBA (14 μM), TMZ (200 μM), or the combination for 7–10 days. Representative spheroid images and quantitative data are shown. (G) Protein expression of GLI1, GLI2, ALDH1, Bmi‐1, CD44, and α‐SMA by Western blot (β‐Actin as loading control). (H) mRNA levels of *CD44*, *Nestin*, *OCT4*, *N‐cadherin* in U87 and U251 cells after the indicated treatment. Data are expressed as mean ± SD. *p < 0.05; **p < 0.01; ***p < 0.001, ^#^ *p* < 0.05; ^##^ *p* < 0.01; ^###^ *p* < 0.001; NS indicates no significant difference between groups.

Functionally, Me‐4‐O‐DBA (14 μM) significantly inhibited glioma cell migration, invasion, and spheroid formation, and the combination with TMZ further enhanced these effects compared to either agent alone (Figure 4D–F). Mechanistically, combined treatment reduced GLI1, Bmi‐1, and ALDH1 protein levels, while Me‐4‐O‐DBA alone more strongly suppressed GLI2, and α‐SMA (Figure 4G). Correspondingly, Nestin, OCT4, and N‐cadherin mRNA levels were also decreased (Figure 4H). These findings suggest that Me‐4‐*O*‐DBA synergises with TMZ to inhibit glioma cell invasion and tumour stemness. Overall, our results propose that the combination of Me‐4‐*O*‐DBA with TMZ holds therapeutic potential for treating malignant gliomas characterised by high invasiveness and stemness.

### 3.5 Me‐4‐*O*‐DBA Combined With TMZ Inhibits Aerobic Glycolysis in Glioma Cells

Since HIF‐1 activation enhances glycolytic metabolic pathways, we examined glucose transporters and glycolysis‐related enzymes [32]. Me‐4‐*O*‐DBA treatment significantly reduced the expression of GLUT1, HK2, and LDHA, along with corresponding HIF‐1α levels under hypoxic conditions in U87 cells (Figure 5A,B). To evaluate the impact of Me‐4‐*O*‐DBA (14 μM), TMZ (200 μM), and their combination (14 μM Me‐4‐*O*‐DBA + 200 μM TMZ) on glycolytic metabolism in glioma cells, we performed Seahorse XF Glycolytic Rate Assays in U87 and U251 cell lines. Treatment with Me‐4‐*O*‐DBA, TMZ, and their combination resulted in alterations in glycolytic parameters, as evidenced by changes in ECAR and OCR measurements, indicating modulation of glycolytic rate (glycoPER) (Figure 5C,D). Combination treatment produced stronger suppression of glycolytic activity than either single treatment. Western blot analysis revealed that the protein levels of GLUT1, HK2, PKM2, and LDHA were significantly decreased in the combination treatment group compared to the control and/or single‐treatment groups (Figure 5E,F). Additionally, qRT‐PCR analysis showed a significant reduction in the mRNA expression levels of GLUT1, HK2, PKM2, and LDHA in U87 and U251 glioma cells following treatment with Me‐4‐*O*‐DBA, TMZ, and their combination compared to the DMSO control (Figure 5G). Overall, our results indicate that the combination of Me‐4‐*O*‐DBA and TMZ effectively suppresses the expression of key glycolytic enzymes in glioma cells, thereby potentially disrupting the Warburg effect and inhibiting the metabolic pathways crucial for glioma cell survival and proliferation.

**FIGURE 5:**
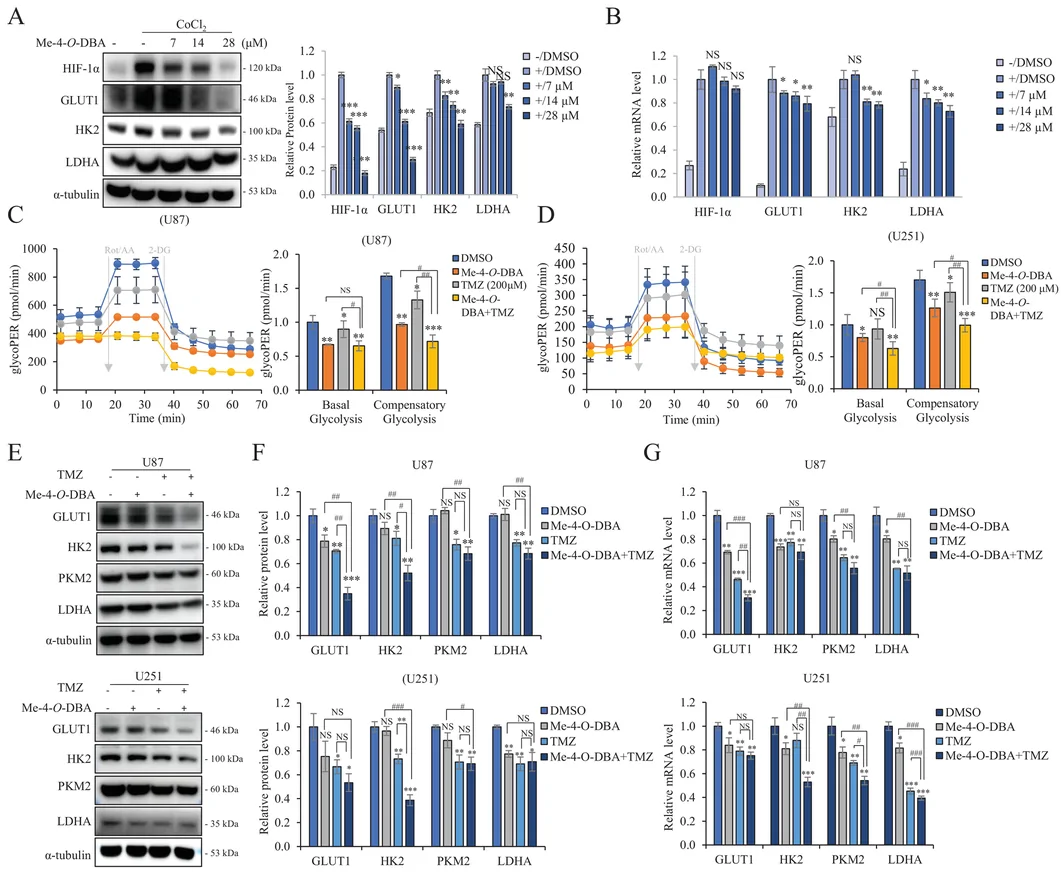
Inhibition of aerobic glycolysis in glioma cells by Me‐4‐*O*‐DBA and TMZ combination. (A) Western blot analysis of HIF‐1α, GLUT1, HK2, and LDHA expression hypoxic conditions (100 μM CoCl_2_ treatment) in U87 cells treated with Me‐4‐*O*‐DBA at the indicated concentrations. α‐tubulin was used as a loading control. Normoxic condition used as control. (B) Quantification of mRNA expression levels of HIF‐1α, GLUT1, HK2, and LDHA following Me‐4‐*O*‐DBA treatment hypoxic conditions. Normoxic condition used as control. (C, D) U87 and U251 glioma cell lines were treated with DMSO, Me‐4‐*O*‐DBA (14 μM), TMZ (200 μM), or 14 μM Me‐4‐*O*‐DBA + 200 μM TMZ for 48 h. The Seahorse XF96 extracellular flux analyser and the Glycolytic Rate Assay Kit were used to measure the oxygen consumption rate (OCR) and extracellular acidification rate (ECAR). These measurements were used to quantify glycolytic activity and determine the glycolytic proton efflux rate (glycoPER). The assay recorded values at two specific time points following sequential injections of rotenone/antimycin A and 2‐DG, providing data on basal and compensatory glycolysis. (E, F) Western blot analysis was performed to examine the expression levels of key metabolic enzymes in response to Me‐4‐*O*‐DBA, TMZ, and their combination. U87 and U251 cells were treated with Me‐4‐*O*‐DBA (14 μM), TMZ (200 μM), or their combination (14 μM Me‐4‐*O*‐DBA + 200 μM TMZ), and protein expression was analysed. (G) Relative mRNA expression levels of key metabolic genes were assessed in U87 and U251 glioma cells following 48 h of treatment with DMSO (control), Me‐4‐*O*‐DBA (14 μM), TMZ (200 μM), or their combination (14 μM Me‐4‐*O*‐DBA + 200 μM TMZ). The expression levels of *GLUT1, HK2, PKM2*, and *LDHA* were determined by qRT‐PCR and normalised to the control group (DMSO‐treated). Data are presented as mean ± SD. *p < 0.05; **p < 0.01; ***p < 0.001, ^#^ *p* < 0.05; ^##^ *p* < 0.01; ^###^ *p* < 0.001; NS indicates no significant difference between groups.

### 3.6 Me‐4‐*O*‐DBA, TMZ, and Their Combination Treatment Induce Broad Inhibition of Mitochondrial Respiration in Glioma Cells

Treatment of glioma cells with Me‐4‐*O*‐DBA (14 μM), TMZ (200 μM), or their combination (14 μM Me‐4‐*O*‐DBA + 200 μM TMZ) led to significant alterations in mitochondrial respiration, as measured by real‐time monitoring of oxidative phosphorylation via the oxygen consumption rate (OCR) using a Seahorse XFe96 analyser. Sequential administration of oligomycin, FCCP, and an antimycin/rotenone mix enabled the evaluation of key mitochondrial parameters, including maximal respiration, basal respiration, ATP production, proton leak, and spare respiratory capacity. Our findings demonstrated a significant reduction in OCR following treatment with Me‐4‐*O*‐DBA, TMZ, and their combination (Figure 6A,B). Additionally, all assessed mitochondrial function parameters exhibited a consistent decline in response to these treatments (Figure 6C), indicating a broad inhibition of mitochondrial respiration in glioma cells. To further explore the molecular mechanisms underlying these effects, we analysed the mRNA expression levels of key regulators of mitochondrial function, including CHD4, PGC‐1α, and PDK1, following treatment with Me‐4‐*O*‐DBA, TMZ, and their combination. In summary, our study highlights the disruptive effects of Me‐4‐*O*‐DBA and TMZ combination therapy on mitochondrial respiration in glioma cells, providing valuable insights into potential therapeutic strategies that target mitochondrial function in glioma treatment.

**FIGURE 6:**
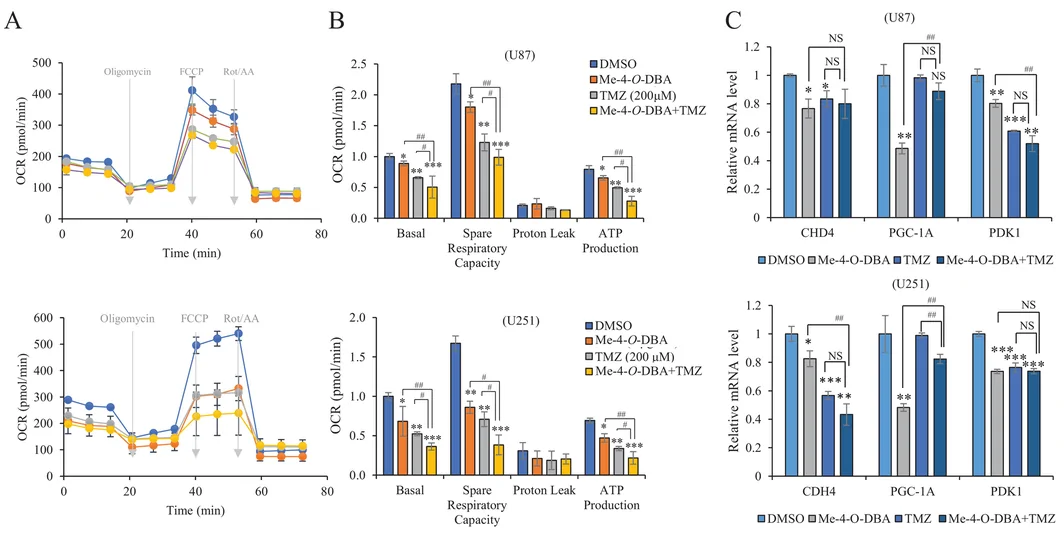
Me‐4‐*O*‐DBA, TMZ, and their combination broadly suppress mitochondrial respiration in glioma cells. (A, B) U87 and U251 glioma cells were treated with Me‐4‐*O*‐DBA (14 μM), TMZ (200 μM), or their combination (14 μM Me‐4‐*O*‐DBA + 200 μM TMZ) for 48 h. Mitochondrial respiration was assessed using the Cell Mito Stress Kit with a Seahorse XF96 Analyser. Oxygen consumption rate (A) and assay parameters (B) such as basal respiration, spare respiratory capacity, proton leak, and ATP production were shown in the figure. (C) To further investigate the effects of Me‐4‐*O*‐DBA, TMZ, and their combination on mitochondrial respiration, qRT‐PCR was performed to analyse the relative mRNA expression levels of *CHD4, PGC‐1α, SRC*, and *PDK1* in U87 and U251 glioma cells after 48 h of treatment. Data is presented as mean ± SD. *p < 0.05; **p < 0.01; ***p < 0.001, ^#^ *p* < 0.05; ^##^ *p* < 0.01; ^###^ *p* < 0.001; NS indicates no significant difference between groups.

### 3.7 Combined Me‐4‐*O*‐DBA and TMZ Treatment Targets HIF‐1α‐Associated Oncogenic Signaling

Comprehensive network analysis revealed that Me‐4‐*O*‐DBA modulates multiple signaling pathways, primarily targeting HIF‐1α (Figure 7A). Western blot analysis further validated these observations, showing significant changes in protein expression levels in response to Me‐4‐*O*‐DBA treatment with or without TMZ treatment. Co‐treatment of Me‐4‐*O*‐DBA and TMZ resulted in stronger inhibitory effects on key oncogenic signaling proteins, including p‐EGFR, t‐EGFR, p‐mTOR, t‐mTOR, p‐Akt, t‐Akt, β‐catenin, cyclin D1, c‐Myc, c‐Fos, and SMAD4, compared to single TMZ or Me‐4‐*O*‐DBA treatment. Interestingly, total JNK (t‐JNK) levels remained unchanged, whereas phosphorylated JNK (p‐JNK) levels were attenuated at high concentrations of Me‐4‐*O*‐DBA combined with TMZ. Additionally, HIF‐1α, c‐Jun, STAT3, Bcl‐xL, and PD‐L1 levels were significantly induced by TMZ treatment. However, Me‐4‐*O*‐DBA potently suppressed TMZ‐induced HIF‐1α and PD‐L1 levels, while also slightly attenuating STAT3 and Bcl‐xL expression (Figure 7B,C). Although the precise mechanism remains unclear, TMZ may contribute to the stabilisation and/or transcriptional induction of HIF‐1α due to inhibition of prolyl hydroxylation and activation of stress‐related pathways [33, 34, 35]. HIF‐1α acts as an adaptive regulator that mediates resistance to TMZ at least in part by maintaining protective autophagy via the control of lysosomes. As previously observed, HIF‐1α knockdown hinders autophagosome maturation by deacidifying the lysosomes due to reduced expression of presenilin‐1 [36, 37]. Moreover, HIF‐1α suppression increases the sensitivity of U251 glioma cells to temozolomide by reducing MGMT expression and blocking activation of the Notch1 signaling pathway [34]. Thus, co‐administration of TMZ with an agent capable of inhibiting HIF‐1α such as Me‐4‐O‐DBA seems to be a reasonable strategy from a mechanistic point of view since it will target this HIF‐1α/lysosomes/autophagy pathway. To identify potential targets of Me‐4‐*O*‐DBA and TMZ in glioma, we conducted an intersection analysis to determine common target genes among Me‐4‐*O*‐DBA, TMZ, and glioma‐related genes. A total of 11 shared target genes were identified, with PIK3CA, GSK3B, and EGFR exhibiting strong protein–protein interactions (Figure 7D). To further investigate PPI network interactions and pathway associations, we performed molecular docking simulations to evaluate the binding affinities of Me‐4‐*O*‐DBA and TMZ with PIK3CA, GSK3B, and EGFR. Me‐4‐*O*‐DBA demonstrated the highest binding affinities, with binding energies of −8.0, −7.7, and −8.2 kcal/mol for PIK3CA, EGFR, and GSK3B, respectively. It formed multiple hydrogen bonds with critical amino acid residues within < 2.2 Å, including ASN‐677, ARG‐705, and SER‐261, indicating strong and stable binding to the active sites of the target proteins. In contrast, TMZ exhibited moderate binding stability, forming hydrogen bonds with residues such as SER‐854, LEU‐703, and ARG‐306, but its binding interactions were weaker than those observed for Me‐4‐*O*‐DBA. These findings suggest that Me‐4‐*O*‐DBA is a potent inhibitor, exhibiting the strongest interaction with the target proteins (Figure 7E). Taken together, these findings indicate that Me‐4‐*O*‐DBA and TMZ co‐treatment effectively regulates oncogenic signaling pathways, mitigates TMZ‐induced adverse effects, and enhances therapeutic efficacy, suggesting a promising strategy for glioma therapy.

**FIGURE 7:**
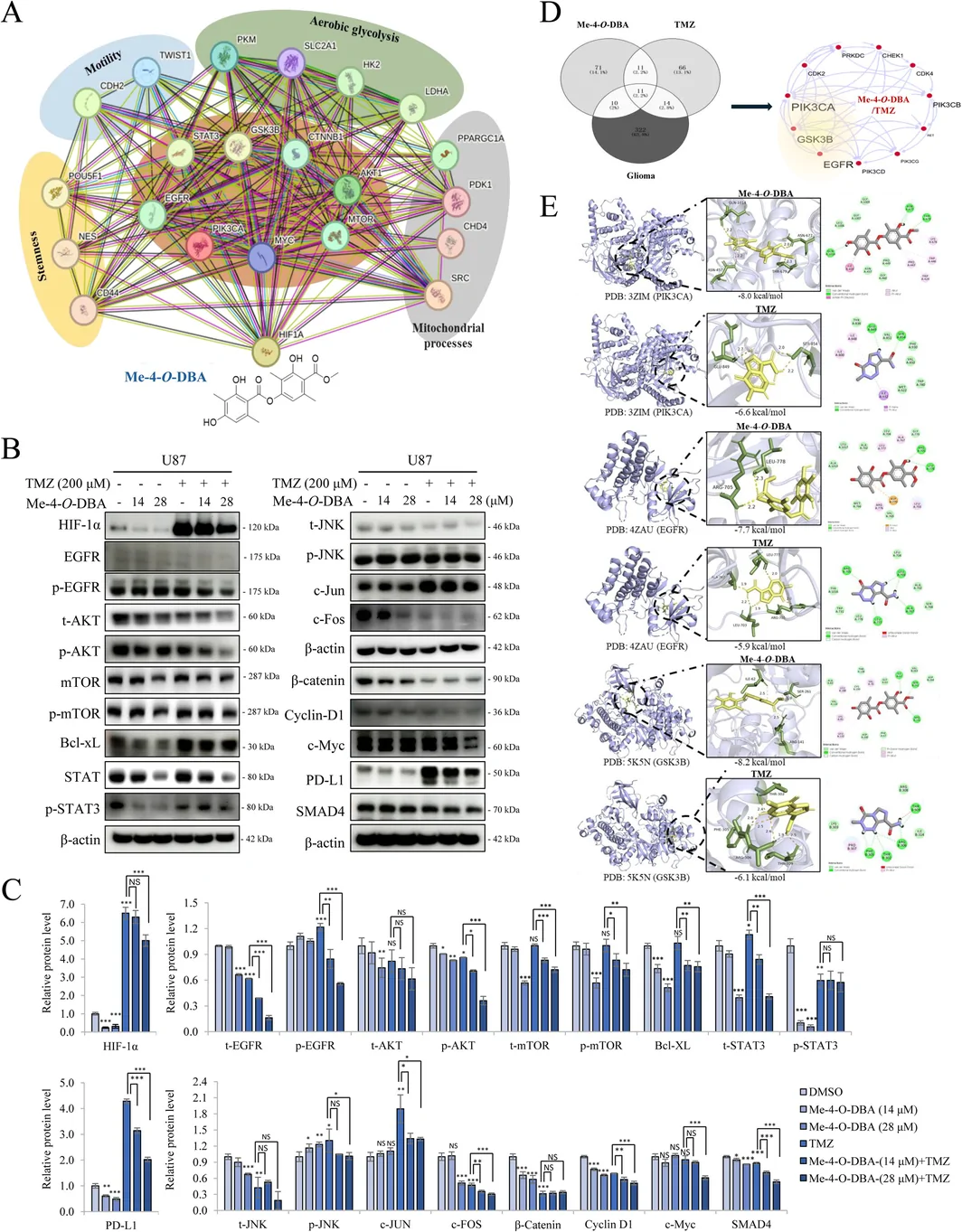
Me‐4‐*O*‐DBA inhibits HIF‐1α accumulation and modulates EMT, stemness, and glycolytic energy metabolism markers. (A) A PPI network analysis of HIF‐1α‐associated proteins using STRING (https://stringdb.org), identifying eight key interacting proteins. (B, C) Suppression of oncogenic signaling by HIF‐1α‐targeted Me‐4‐*O*‐DBA, involving stemness, cell motility, aerobic glycolysis, mitochondrial respiration, and cellular signaling. U87 cells were treated with Me‐4‐*O*‐DBA (14 or 28 μM) and TMZ (200 μM) alone or in combination for 48 h. The total protein was immunoblotted with the indicated antibodies. β‐Actin served as a loading control. Data are presented as mean ± SD. *p < 0.05; **p < 0.01; ***p < 0.001; NS indicates no significant difference between groups. (D) Network pharmacology of Me‐4‐*O*‐DBA and TMZ in glioma treatment. Identification of common target genes: The 11 intersecting target genes associated with Me‐4‐*O‐*DBA, TMZ, and glioma were identified. PPI analysis highlights PIK3CA, GSK3B, and EGFR as key targets within their interaction networks. (E) Molecular docking of Me‐4‐*O*‐DBA and TMZ with PIK3CA (PDB: 3ZIM), EGFR (PDB: 4ZAU), and GSK3B (PDB: 5K5N), showing their binding interactions.

### 3.8 Evaluation of Solubility, Physical Properties and Toxicity of Me‐4‐*O*‐DBA

The physicochemical, pharmacokinetic, and toxicity profiles of Me‐4‐*O*‐DBA and atranorin were evaluated to assess their potential bioavailability, tissue distribution, and therapeutic applicability. The water‐solubility values of Me‐4‐*O*‐DBA and atranorin are similarly small. However, in terms of Caco2 permeability and human intestinal absorption, Me‐4‐*O*‐DBA gave an absorption rate of 71.98%, relative to the 62.87% obtained for atranorin. Since neither compounds are predicted to be P‐gp substrates but not inhibitors, no active transport/inhibitory effects via P‐gp would be expected; they are also similar regarding their volumes of distribution. In metabolic clearance, both compounds have a low level of metabolism via CYP450; Me‐4‐*O*‐DBA is not an substrate of the CYP2D6 and CYP3A4 enzymes but is an inhibitor of the CYP2C19, CYP2C9 and CYP3A4. When the excretion profiles are considered, the total clearance values become comparable; atranorin excretes slightly higher with 0.846 mL/min/kg. Neither compound presents a risk of hepatotoxicity (Table 1). To address the drug distribution of Me‐4‐*O*‐DBA, we examined its distribution in brain tissue and plasma samples after mice treated with control (DMSO), Me‐4‐*O*‐DBA (5, 10, and 20 mg/kg) for 2 weeks, every other day of treatment. Taken together, Me‐4‐*O*‐DBA was distributed within brain and plasma tissues, 10 mg/kg giving optimum results (Figure S4A,B).

**TABLE 1:** Physicochemical properties and ADME predictions of Me‐4‐*O*‐DBA I and atranorin.

Properties	Parameters	Me‐4‐*O*‐DBA	Atranorin
Identity information	PubChem CID	10451136	68066
Absorption	Water solubility	−3.801 (log mol/L)	−4.019 (log mol/L)
	Caco2 permeability	1.058 (log Papp in 10–6 cm/s)	1.038 (log Papp in 10–6 cm/s)
	Intestinal absorption (human)	71.989 (% absorbed)	62.877 (% absorbed)
	Skin permeability	−2.737 (log Kp)	−2.735 (log Kp)
	P‐glycoprotein substrate	Yes	Yes
	P‐glycoprotein I inhibitor	No	No
	P‐glycoprotein II inhibitor	No	No
Distribution	VDss (human)	−0.093 (log L/kg)	−0.26 (log L/kg)
	Fraction unbound (human)	0.174 (Fu)	0.212 (Fu)
	BBB permeability	−1.271 (log BB)	−1.364 (log BB)
	CNS permeability	−3.322 (log PS)	−3.383 (log PS)
Metabolism	CYP2D6 substrate	No	No
	CYP3A4 substrate	No	No
	CYP1A2 inhibitior	No	No
	CYP2C19 inhibitior	Yes	No
	CYP2C9 inhibitior	Yes	No
	CYP2D6 inhibitior	No	No
	CYP3A4 inhibitior	Yes	Yes
Excretion	Total clearance	0.83 (log mL/min/kg)	0.846 (log mL/min/kg)
	Renal OCT2 substrate	No	No
Toxicity	AMES toxicity	Yes	No
	Max. tolerated dose (human)	1.038 (log mg/kg/day)	1.182 (log mg/kg/day)
	hERG I inhibitor	No	No
	hERG II inhibitor	Yes	No
	Oral rat acute toxicity (LD50)	2.433 (mol/kg)	2.445 (mol/kg)
	Oral rat chronic toxicity (LOAEL)	1.634 (log mg/kg_bw/day)	1.658 (log mg/kg_bw/day)
	Hepatotoxicity	No	No
	Skin sensitisation	No	No
	*T. pyriformis* toxicity	0.31 (log μg/L)	0.287 (log μg/L)
	Minnow toxicity	−0.2 (log mM)	0.02 (log mM)

### 3.9 Me‐4‐*O*‐DBA Enhances the Temozolomide Effect on U87‐Derived Tumour‐Bearing Zebrafish Model

We developed a quantitative in vivo cell proliferation assay to evaluate cellular responses to anti‐cancer agents. To assess the cytotoxic effects of Me‐4‐*O*‐DBA and its combination with TMZ on zebrafish embryogenesis, embryos at 6 hpf were exposed to various concentrations of Me‐4‐*O*‐DBA (3, 6, and 14 μM), either alone or in combination with 200 μM TMZ. At 72 hpf, larvae treated with 14 μM Me‐4‐*O*‐DBA, alone or combined with TMZ, exhibited noticeable malformations compared to the DMSO control group, including reduced body length and pericardial edema. In contrast, embryos treated with 3 or 6 μM Me‐4‐*O*‐DBA displayed no obvious phenotypic abnormalities up to 72 hpf, indicating that these lower concentrations were non‐toxic to zebrafish embryonic development (Figure 8A,B). We injected fluorescently labelled human U87 cells into the brain ventricle of 48 hpf zebrafish larvae. To evaluate the in vivo effects of Me‐4‐*O*‐DBA alone or in combination with temozolomide (TMZ), U87 cells were treated with Me‐4‐*O*‐DBA (3 and 6 μM), either alone or combined with 200 μM TMZ, following inoculation (Figure 8C). Treatment with 6 μM Me‐4‐*O*‐DBA resulted in a reduction of tumour area, with a more pronounced, dose‐dependent decrease observed when combined with TMZ (Figure 8D,E). Notably, tumour fluorescence intensity was significantly diminished in larvae treated with the combination of 6 μM Me‐4‐*O*‐DBA and 200 μM TMZ. These findings further support the therapeutic potential of Me‐4‐*O*‐DBA, particularly in combination with TMZ, for inhibiting tumour growth in vivo.

**FIGURE 8:**
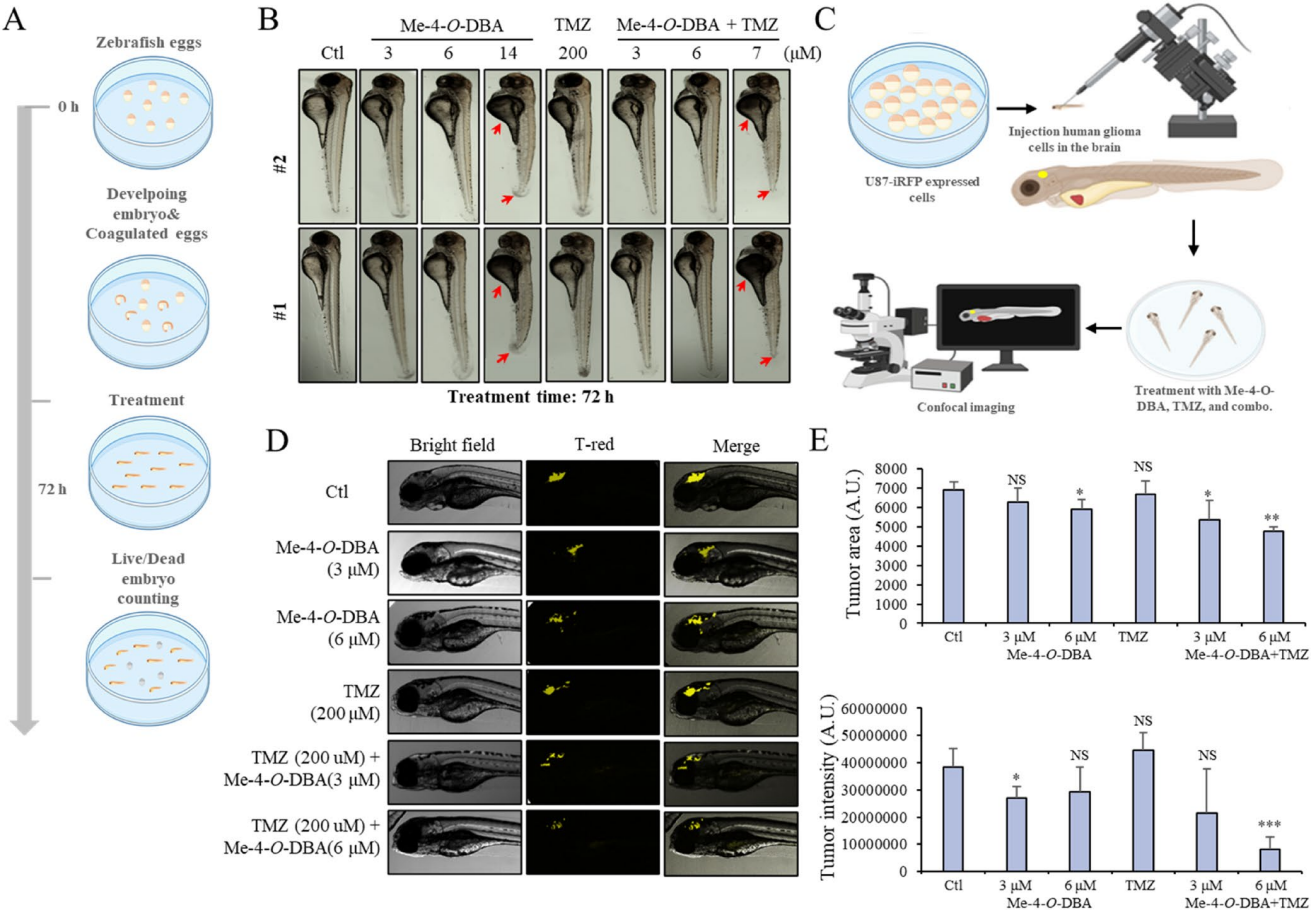
Me‐4‐*O*‐DBA in combination with TMZ enhances the therapeutic effect in U87‐derived tumour‐bearing zebrafish model. (A) Diagram illustrating the morphological effects of zebrafish larvae exposure to various concentrations of Me‐4‐*O*‐DBA (3, 6, and 14 μM), either alone or in combination with 200 μM TMZ at 72 hpf. (B) 14 μM Me‐4‐*O*‐DBA induced a developmental deficiency in treated embryos at 72 hpf; a shorter body length and pericardial edema were observed (*n* = 24). Scale bar: 300 μm. (C) Schematic representation of the transplantation of fluorescently labelled human U87 cells into the brain ventricle of zebrafish embryos. The tumour was allowed to engraft for 24 h before being divided into control and treatment groups. Treatments containing 3 and 6 μM of Me‐4‐*O*‐DBA, either alone or in combination with 200 μM TMZ, were diluted in PTU solution. (D) Representative images for DMSO, 3 or 6 μM of Me‐4‐*O*‐DBA, and combinatorial treatment with TMZ, along with corresponding quantitative analyses of tumour area and tumour intensity (*n* = 3). Scale bar: 300 μm. (E) Data represents the mean ± SD. *p < 0.05; **p < 0.01; ***p < 0.001; NS: No significant difference compared with DMSO‐treated tumours.

## 4 Discussion

Lichen secondary metabolites have demonstrated significant anticancer potential in various studies. These compounds, including polyketides, depsides, depsidones, usnic acid, and quinones, exhibit cytotoxic, pro‐apoptotic, and anti‐proliferative effects on cancer cells [38, 39]. Among them, depsides have shown particularly promising anticancer activity. Gyrophoric acid, a common depside, has been found to exert cytotoxic effects against breast cancer (MCF‐7) and colon cancer (HT‐29) cells [40]. Lecanoric acid promotes apoptosis by increasing reactive oxygen species (ROS) levels and activating caspases, which are essential for programmed cell death [41]. Literature findings demonstrate that among the tested lichen secondary metabolites, olivetoric acid (OLA) exhibited the strongest cytotoxic effect on GBM cells, followed by psoromic acid (PSA) and physodic acid (PHA), with IC_50_ values indicating a selective impact on cancerous cells. Moreover, the observed cytotoxicity was positively correlated with increased LDH activity and oxidative DNA damage, suggesting that these metabolites may exert their antitumour effects through oxidative stress‐mediated mechanisms [42]. Another study investigated the impact of diffractaic acid (DA), lobaric acid (LA), and (+)‐usnic acid (UA) on cell proliferation, oxidative balance, and DNA damage in human U87MG‐GBM cells and primary rat cerebral cortex (PRCC) cells. The findings indicated that both cell types exhibited a dose‐dependent rise in lactate dehydrogenase activity and oxidative DNA damage, as measured by 8‐hydroxy‐2′‐deoxyguanosine (8‐OH‐dG) levels. Additionally, IC_50_ analysis identified LA as the most cytotoxic compound in GBM cells (5.77 mg/L), whereas DA and UA demonstrated significant antioxidant effects in PRCC cells at a concentration of 10 mg/L [43]. Another lichen secondary metabolite, 7β‐22 dihydroxyhopane (AP 18), significantly reduced glioma stem cell (GSC) viability and proliferation while inducing apoptosis, as confirmed by Annexin V/PI staining and 3‐D optical diffraction tomography (ODT) imaging. Additionally, AP 18 suppressed GSC stemness, suggesting its potential as a therapeutic agent for targeting glioblastoma stem cells [44]. Atranorin, another lichen depside, has been reported to induce G1 phase arrest in liver cancer cells, thereby inhibiting uncontrolled cell proliferation [20]. Additionally, atranorin reduces cancer cell migration and invasion, effectively preventing metastasis [22]. Lichen secondary metabolites are increasingly being explored as potential candidates for novel cancer therapies. Here, we investigated the inhibitory effects of Me‐4‐*O*‐DBA, an atranorin derivative, on glioma cell lines. Our major findings include: (1) Me‐4‐*O*‐DBA suppressed glioma cell invasion, spheroid formation, and proliferation by downregulating EMT, cancer stemness markers, and energy metabolism; (2) molecular docking results revealed Me‐4‐*O*‐DBA specifically targets HIF‐1α at Lys‐304, a known ubiquitin acceptor site, and facilitates its ubiquitination‐dependent trafficking and degradation; (3) Me‐4‐*O*‐DBA, in combination with temozolomide (TMZ), a standard chemotherapeutic drug for GBM patients, effectively inhibited glioma cell motility and spheroid formation; (4) Me‐4‐*O*‐DBA and TMZ synergistically targeted PI3KCA, EGFR, and GSK3β, regulating more oncogenic signaling pathways and mitigating TMZ‐induced adverse effects; (5) zebrafish models further validated the therapeutic potential of Me‐4‐*O*‐DBA in glioma treatment. These results suggest that Me‐4‐*O*‐DBA has significant pharmaceutical potential for the treatment of glioma.

Hypoxia is one of the main microenvironmental factors that promote tumour progression in glioma and HIF‐1α plays a critical role in this process. HIF‐1α regulates processes such as EMT, stemness, cell motility and metabolic reprogramming in glioma cells by stabilising in hypoxic environments [45, 46, 47]. In particular, it increases the expression of EMT factors such as Snail, Slug, TWIST and ZEB1/2, making cells more invasive [48]. Furthermore, HIF‐1α activates transcription factors such as OCT4, SOX2 and NANOG, which promotes the survival of glioma stem‐like cells (GSCs) and contributes to the development of resistance to therapy [49, 50]. At the metabolic level, HIF‐1α promotes the Warburg effect by increasing the expression of glycolytic enzymes such as GLUT1 and HK2 and increases the dependence of glioma cells on anaerobic glycolysis [51, 52, 53]. HIF‐1α interacts bidirectionally with key signaling pathways such as EGFR, PI3K/AKT/mTOR and GSK3β in glioma. EGFR activation increases HIF‐1α stability, creating a positive feedback mechanism that enhances hypoxic signaling. Similarly, the PI3K/AKT/mTOR signaling pathway promotes hypoxic adaptation by increasing the protein stability of HIF‐1α and increases proliferation in glioma cells [54, 55]. Activation of HIF‐1α, together with genetic alterations such as loss of PTEN, may activate the mTORC1 pathway and cause gliomas to acquire an aggressive phenotype [56, 57, 58]. On the other hand, GSK3β, normally involved in proteasomal degradation of HIF‐1α, is inhibited under hypoxia and increases the stability of HIF‐1α, which promotes EMT and maintenance of stem cell phenotype [59, 60]. Given all these mechanisms, targeting HIF‐1α may be a promising strategy in the treatment of glioma. Our findings suggest that Me‐4‐*O*‐DBA enhances the degradation of HIF‐1α and downregulates associated target genes and transcriptional regulators. Future studies may contribute to the development of new therapeutic approaches by further investigating the interactions between HIF‐1α and upstream and downstream signaling pathways.

GBM is one of the most aggressive and treatment‐resistant brain tumours. As monotherapy has proven ineffective, combination therapies are becoming increasingly important in treating refractory diseases like GBM [61]. TMZ is the standard‐of‐care chemotherapy, but its effectiveness is often limited due to resistance. Combination treatments involving TMZ with other agents are crucial for improving patient outcomes [62]. Lots of evidence showed that the combination of some drugs with TMZ enhanced the antitumour capacity of TMZ in human glioma cells, such as lomustine [63], demethoxycurcumin [64], and perifosine [65, 66]. Specifically, certain lichen secondary metabolites have been found to inhibit the Wnt/β‐catenin pathway in glioblastoma cells, a signaling pathway often associated with tumour progression and resistance to therapy. By targeting this pathway, these metabolites not only suppress tumour growth but also improve the anticancer effects of temozolomide, a standard chemotherapeutic drug used in glioblastoma treatment [21]. In this study, we found that Me‐4‐*O*‐DBA acted synergistically (CI < 1.0) with TMZ to inhibit the cell viability of gliomas (Figure 4A). Moreover, our results demonstrate that the combination of Me‐4‐*O*‐DBA and TMZ inhibited cell motility, spheroid formation, and proliferation in glioma cells. Based on these findings, we suggest that combining Me‐4‐*O*‐DBA with TMZ may be a promising approach for treating malignant gliomas with metastasis and stem‐like properties, as targeting multiple factors involved in glioma cell growth could enhance the therapeutic effect and decrease the risk of resistance. Our results showed that HIF‐1α, c‐Jun, STAT3, Bcl‐xL, and PD‐L1 levels were significantly induced by TMZ treatment (Figure 7B), indicating that TMZ triggers a defensive response in tumour cells, potentially leading to chemotherapy resistance through mechanisms related to inflammation, hypoxia, and survival. However, Me‐4‐*O*‐DBA effectively suppressed TMZ‐induced HIF‐1α and PD‐L1 levels (key factors in tumour survival and immune evasion), while also modestly reducing STAT3 and Bcl‐xL expression. Supporting our in vitro findings, the zebrafish xenograft model confirmed the antitumour activity of Me‐4‐*O*‐DBA. Treatment with 6 μM Me‐4‐*O*‐DBA, especially in combination with TMZ, significantly reduced tumour area and fluorescence intensity without inducing developmental toxicity at lower doses. In vivo results reinforce the therapeutic relevance of Me‐4‐*O*‐DBA and highlight its potential in combinatorial glioma therapy. Overall findings suggest that the combination of Me‐4‐*O*‐DBA and TMZ enhances therapeutic efficacy and overcomes TMZ resistance (Figure 9).

**FIGURE 9:**
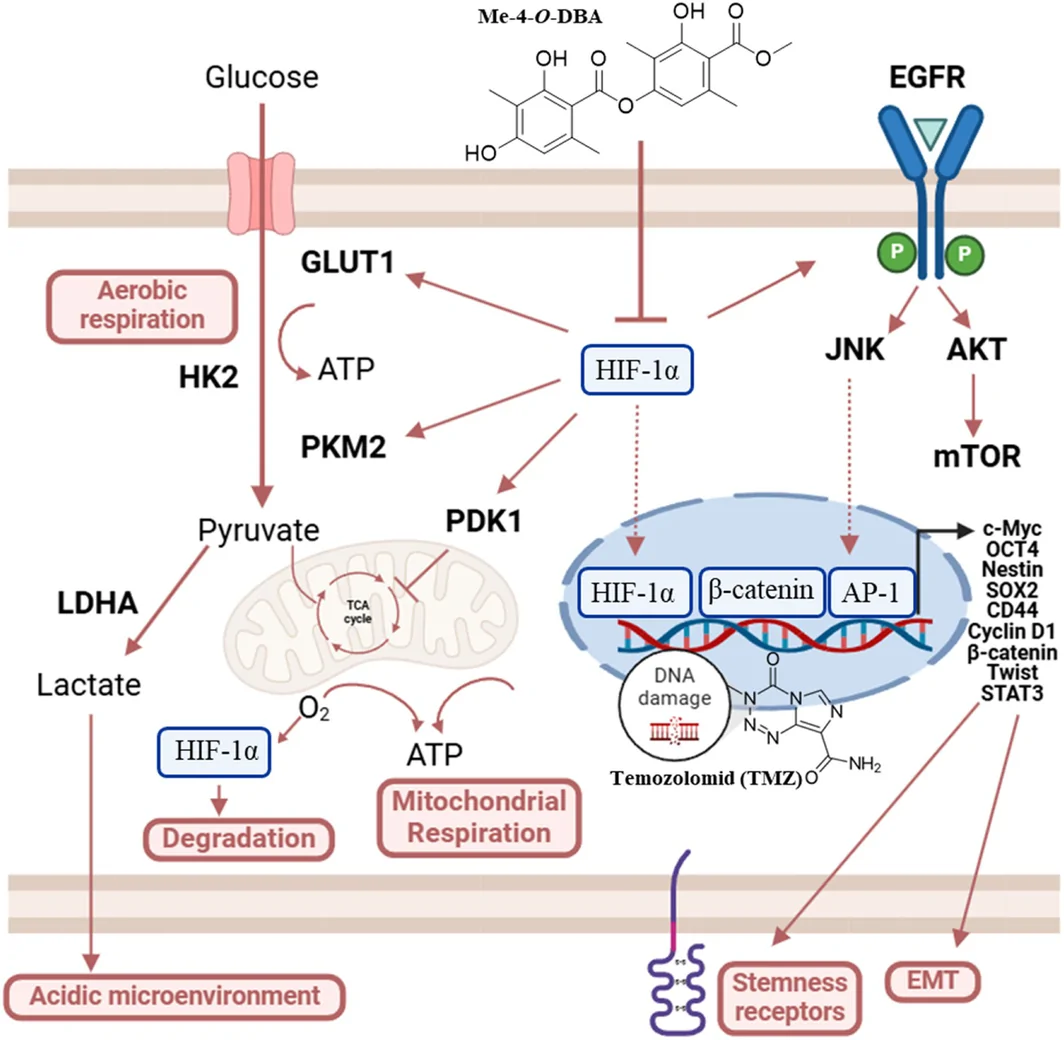
Schematically showing that the combined treatment of Me‐4‐*O*‐DBA and TMZ blocks glioma progression. Glucose metabolism is upregulated through key glycolytic enzymes, including HK2, PKM2, and PDK1, leading to ATP production and lactate accumulation, which creates an acidic microenvironment by HIF‐1α. HIF‐1α is stabilised under hypoxic conditions, enhancing tumour progression via β‐catenin and AP‐1, promoting stemness‐associated markers (c‐Myc, OCT4, Nestin, SOX2, CD44) and EMT. EGFR signaling through JNK and AKT/mTOR further activates HIF‐1α, supporting glioma cell survival and resistance to therapies like Temozolomide (TMZ).

In recent years, zebrafish models have gained increasing popularity as an alternative in vivo platform due to their cost‐effectiveness, ease of genetic manipulation, and suitability for high‐throughput screening—especially under the limitations of traditional rodent models in terms of technical complexity, cost, and group size constraints [67]. Although intracranial implantation in mouse orthotopic glioma models allows for anatomically accurate tumour localisation, the procedure requires exceptional technical precision. Even slight deviations in injection coordinates, volume, or rate can result in variability across experimental groups or cause unintended tissue damage [68]. Despite these challenges, orthotopic mouse models remain critical for evaluating glioma invasiveness and treatment responses in a physiologically relevant context. Therefore, to further validate the therapeutic efficacy of Me‐4‐*O*‐DBA and its analogs, subsequent studies should include in vivo assessments using rodent orthotopic glioma models.

## 5 Conclusion

In conclusion, our findings indicate that Me‐4‐*O*‐DBA exhibits significant anticancer activities against glioma cells in vitro and in vivo. Me‐4‐*O*‐DBA could be developed as a targeted therapy against HIF‐1α, effectively suppressing malignant behaviours in glioma. Me‐4‐*O*‐DBA suppressed glioma cell motility, stemness, and energy metabolism via the inhibition of multiple developmental signaling pathways, suggesting that Me‐4‐*O*‐DBA has great potential for development as an inhibitor against invasiveness and stemness in gliomas. Furthermore, Me‐4‐*O*‐DBA in combination with TMZ enhances the inhibitory effect and reduces the potential therapeutic resistance. Further research into monotherapy or combined inhibition strategies may lead to more effective treatments for glioma patients, offering potential to overcome resistance and improve therapeutic outcomes.

## Author Contributions

R.Z., M.V. and H.K. conceived and designed the experiments. R.Z. and M.V. contributed equally to this work and shared first authorship. R.Z. and M.V. performed the chemical analysis, bioassays, analysed the data and wrote the manuscript. H.‐H.H., J.‐S.H. and S.K.K. contributed materials and analysis tools. H.P. contributed to in vivo experiment. J.Y. and R.L. contributed to the chemical analysis experiments. All data were generated in‐house, and no paper mill was used. All authors agree to be accountable for all aspects of work ensuring integrity and accuracy.

## Funding

This work was supported by the National Research Foundation of Korea (NRF) grant funded by the Korea government (MSIT) (RS‐2024‐00413760) and the BK21 FOUR (Fostering Outstanding Universities for Research) program funded by the Ministry of Education (MOE, Korea) (No. 2120260515565).

## Conflicts of Interest

The authors declare no conflicts of interest.

## Supporting information


**Figure S1:** ^1^H NMR spectrum of methyl 4‐*O*‐demethylbarbatate (Me‐4‐*O*‐DBA) in DMSO.
**Figure S2:** ^1^H NMR spectrum of methyl 4‐*O*‐demethylbarbatate (Me‐4‐*O*‐DBA) in DMSO, D_2_O.
**Figure S3:** ESIMS spectrum of Me‐4‐O‐DBA.
**Figure S4:** Distribution of Me‑4‑O‑DBA following intraperitoneal injections at doses of 5, 10, or 20 mg/kg every other day for 2 weeks. Quantitative LC–MS/MS analysis of Me‑4‑O‑DBA levels in the plasma (A) and brain (B) of C57BL/6 mice.
**Table S1:** Primer sequences for qRT‐PCR.
**Table S2:** Antibody information.

## Data Availability

The data that support the findings of this study are available from the corresponding author upon reasonable request.

## References

[1] A. C. Tan , D. M. Ashley , G. Y. López , M. Malinzak , H. S. Friedman , and M. Khasraw , “Management of Glioblastoma: State of the Art and Future Directions,” CA: A Cancer Journal for Clinicians 70 (2020): 299–312, 10.3322/CAAC.21613. 32478924

[2] L. A. D. Cooper , D. A. Gutman , C. Chisolm , et al., “The Tumor Microenvironment Strongly Impacts Master Transcriptional Regulators and Gene Expression Class of Glioblastoma,” American Journal of Pathology 180 (2012): 2108–2119, 10.1016/J.AJPATH.2012.01.040. PMC3354586 22440258

[3] D. Uribe , Á. Torres , J. D. Rocha , et al., “Multidrug Resistance in Glioblastoma Stem‐Like Cells: Role of the Hypoxic Microenvironment and Adenosine Signaling,” Molecular Aspects of Medicine 55 (2017): 140–151, 10.1016/J.MAM.2017.01.009. 28223127

[4] A. J. Yool and S. Ramesh , “Molecular Targets for Combined Therapeutic Strategies to Limit Glioblastoma Cell Migration and Invasion,” Frontiers in Pharmacology 11 (2020): 515819, 10.3389/FPHAR.2020.00358. PMC7118801 32292341

[5] R. Ma , M. J. B. Taphoorn , and P. Plaha , “Advances in the Management of Glioblastoma,” Journal of Neurology, Neurosurgery, and Psychiatry 92 (2021): 1103–1111, 10.1136/JNNP-2020-325334. 34162730

[6] H. D. Thanh , S. Lee , T. T. Nguyen , et al., “Temozolomide Promotes Matrix Metalloproteinase 9 Expression Through p38 MAPK and JNK Pathways in Glioblastoma Cells,” Scientific Reports 14, no. 1 (2024): 1–13, 10.1038/s41598-024-65398-2. PMC11192740 38906916

[7] J. P. Thiery , H. Acloque , R. Y. J. Huang , and M. A. Nieto , “Epithelial‐Mesenchymal Transitions in Development and Disease,” Cell 139 (2009): 871–890, 10.1016/J.CELL.2009.11.007. 19945376

[8] S. A. Mani , W. Guo , M. J. Liao , et al., “The Epithelial‐Mesenchymal Transition Generates Cells With Properties of Stem Cells,” Cell 133 (2008): 704–715, 10.1016/J.CELL.2008.03.027. PMC2728032 18485877

[9] M. Varlı , E. Y. Lee , Y. Yang , et al., “1′‐O‐Methyl‐Averantin Isolated From the Endolichenic Fungus Jackrogersella sp. EL001672 Suppresses Colorectal Cancer Stemness via Sonic Hedgehog and Notch Signaling,” Scientific Reports 13, no. 1 (2023): 1–14, 10.1038/s41598-023-28773-z. PMC9935543 36797277

[10] N. N. Pavlova and C. B. Thompson , “The Emerging Hallmarks of Cancer Metabolism,” Cell Metabolism 23 (2016): 27–47, 10.1016/J.CMET.2015.12.006. PMC4715268 26771115

[11] W. Luo , Z. Xu , H. Wang , et al., “HIF1A‐Repressed PUS10 Regulates NUDC/Cofilin1 Dependent Renal Cell Carcinoma Migration by Promoting the Maturation of miR‐194‐5p,” Cell & Bioscience 13 (2023): 1–20, 10.1186/S13578-023-01094-4. PMC10439626 37596681

[12] Q. Su , J. Wang , Q. Wu , et al., “Sanguinarine Combats Hypoxia‐Induced Activation of EphB4 and HIF‐1α Pathways in Breast Cancer,” Phytomedicine 84 (2021): 153503, 10.1016/J.PHYMED.2021.153503. 33636580

[13] M. Varlı , S. R. Bhosle , E. Kim , et al., “Usnic Acid Targets 14‐3‐3 Proteins and Suppresses Cancer Progression by Blocking Substrate Interaction,” JACS Au 4 (2024): 1521–1537, 10.1021/JACSAU.3C00774. PMC11040559 38665668

[14] E. E. Wicks and G. L. Semenza , “Hypoxia‐Inducible Factors: Cancer Progression and Clinical Translation,” Journal of Clinical Investigation 132 (2022): e159839, 10.1172/JCI159839. PMC9151701 35642641

[15] W. R. Wilson and M. P. Hay , “Targeting Hypoxia in Cancer Therapy,” Nature Reviews Cancer 11, no. 6 (2011): 393–410, 10.1038/nrc3064. 21606941

[16] C. D. B. Gamage , J. H. Kim , Y. Yang , et al., “Libertellenone T, a Novel Compound Isolated From Endolichenic Fungus, Induces G2/M Phase Arrest, Apoptosis, and Autophagy by Activating the ROS/JNK Pathway in Colorectal Cancer Cells,” Cancers 15 (2023): 489, 10.3390/CANCERS15020489/S1. PMC9857212 36672439

[17] A. Galanty , P. Koczurkiewicz , D. Wnuk , et al., “Usnic Acid and Atranorin Exert Selective Cytostatic and Anti‐Invasive Effects on Human Prostate and Melanoma Cancer Cells,” Toxicology In Vitro 40 (2017): 161–169, 10.1016/J.TIV.2017.01.008. 28095330

[18] A. Harikrishnan , V. Veena , B. Lakshmi , et al., “Atranorin, an Antimicrobial Metabolite From Lichen Parmotrema Rampoddense Exhibited In Vitro Anti‐Breast Cancer Activity Through Interaction With Akt Activity,” Journal of Biomolecular Structure & Dynamics 39 (2021): 1248–1258, 10.1080/07391102.2020.1734482. 32096436

[19] G. g. Huang , H. y. Wang , X. h. Wang , et al., “Atranorin Inhibits Zika Virus Infection in Human Glioblastoma Cell Line SNB‐19 via Targeting Zika Virus Envelope Protein,” Phytomedicine 125 (2024): 155343, 10.1016/J.PHYMED.2024.155343. 38290230

[20] Y. J. Jeon , S. Kim , J. H. Kim , U. J. Youn , and S. S. Suh , “The Comprehensive Roles of ATRANORIN, A Secondary Metabolite From the Antarctic Lichen Stereocaulon Caespitosum, in HCC Tumorigenesis,” Molecules 24 (2019): 1414, 10.3390/MOLECULES24071414. PMC6480312 30974882

[21] A. Majchrzak‐Celińska , R. Kleszcz , E. Studzińska‐Sroka , et al., “Lichen Secondary Metabolites Inhibit the Wnt/β‐Catenin Pathway in Glioblastoma Cells and Improve the Anticancer Effects of Temozolomide,” Cells 11 (2022): 1084, 10.3390/CELLS11071084. PMC8997913 35406647

[22] R. Zhou , Y. Yang , S. Y. Park , et al., “The Lichen Secondary Metabolite Atranorin Suppresses Lung Cancer Cell Motility and Tumorigenesis,” Scientific Reports 7, no. 1 (2017): 1–13, 10.1038/s41598-017-08225-1. PMC5557893 28811522

[23] W. Kim , R. Liu , S. Woo , et al., “Linking a Gene Cluster to Atranorin, a Major Cortical Substance of Lichens, Through Genetic Dereplication and Heterologous Expression,” MBio 12 (2021): 10–128, 10.1128/MBIO.01111-21/SUPPL_FILE/MBIO.01111-21-ST002.DOCX. PMC8262933 34154413

[24] M. Varlı , R. Zhou , R. Liu , et al., “4‐O‐Demethylbarbatic Acid Has a Novel Therapeutic Impact on the Progression of EGFR‐Driven Lung Adenocarcinoma,” Phytomedicine 145 (2025): 156973, 10.1016/J.PHYMED.2025.156973. 40578034

[25] S. R. M. Ibrahim , A. Sirwi , B. G. Eid , S. G. A. Mohamed , and G. A. Mohamed , “Fungal Depsides‐Naturally Inspiring Molecules: Biosynthesis, Structural Characterization, and Biological Activities,” Metabolites 11 (2021): 683, 10.3390/METABO11100683. PMC8540757 34677398

[26] T. C. Chou and N. Martin , CompuSyn for Drug Combinations: PC Software and User's Guide: A Computer Program for Quantitation of Synergism and Antagonism in Drug Combinations, and the Determination of IC50 and ED50 and LD50 Values (ComboSyn, 2005).

[27] M. Vittori , H. Motaln , and T. L. Turnšek , “The Study of Glioma by Xenotransplantation in Zebrafish Early Life Stages,” Journal of Histochemistry and Cytochemistry 63 (2015): 749–761, 10.1369/0022155415595670. PMC4823804 26109632

[28] Y. V. Liu , J. H. Baek , H. Zhang , R. Diez , R. N. Cole , and G. L. Semenza , “RACK1 Competes With HSP90 for Binding to HIF‐1α and Is Required for O2‐Independent and HSP90 Inhibitor‐Induced Degradation of HIF‐1α,” Molecular Cell 25 (2007): 207–217, 10.1016/j.molcel.2007.01.001. PMC2563152 17244529

[29] Y. V. Liu and G. L. Semenza , “RACK1 vs. HSP90: Competition for HIF‐1α Degradation vs. Stabilization,” Cell Cycle 6 (2007): 656–659, 10.4161/CC.6.6.3981. 17361105

[30] D. J. Klionsky , Z. Elazar , P. O. Seglen , and D. C. Rubinsztein , “Does Bafilomycin A1 Block the Fusion of Autophagosomes With Lysosomes?,” Autophagy 4 (2008): 849–850, 10.4161/AUTO.6845. 18758232

[31] M. Mauthe , I. Orhon , C. Rocchi , et al., “Chloroquine Inhibits Autophagic Flux by Decreasing Autophagosome‐Lysosome Fusion,” Autophagy 14 (2018): 1435–1455, 10.1080/15548627.2018.1474314. PMC6103682 29940786

[32] T. Duraj , N. García‐romero , J. Carrión‐navarro , et al., “Beyond the Warburg Effect: Oxidative and Glycolytic Phenotypes Coexist Within the Metabolic Heterogeneity of Glioblastoma,” Cells 10 (2021): 202, 10.3390/CELLS10020202. PMC7922554 33498369

[33] X. Li , G. Yu , X. Zhong , et al., “P4HA1 Mediates YAP Hydroxylation and Accelerates Collagen Synthesis in Temozolomide‐Resistant Glioblastoma,” Chinese Medical Journal 138 (2025): 1991–2005, 10.1097/CM9.0000000000003679. PMC12369758 40484999

[34] J. H. Tang , Z. X. Ma , G. H. Huang , et al., “Downregulation of HIF‐1a Sensitizes U251 Glioma Cells to the Temozolomide (TMZ) Treatment,” Experimental Cell Research 343 (2016): 148–158, 10.1016/J.YEXCR.2016.04.011. 27090014

[35] J. S. Thelwell and A. J. Johnson , “Activation of the HIF1α Pathway in Neurologic Disease: A Targetable Master Regulator to Reduce Neuropathology,” 7 (2026): 18, 10.20944/PREPRINTS202605.0932.V1.

[36] Z. Kun , Y. Yuling , W. Dongchun , X. Bingbing , L. Xiaoli , and X. Bin , “HIF‐1α Inhibition Sensitized Pituitary Adenoma Cells to Temozolomide by Regulating Presenilin 1 Expression and Autophagy,” Technology in Cancer Research & Treatment 15 (2016): NP95–NP104, 10.1177/1533034615618834. 26647409

[37] N. Singh , A. Miner , L. Hennis , and S. Mittal , “Mechanisms of Temozolomide Resistance in Glioblastoma—A Comprehensive Review,” Cancer Drug Resistance 4 (2021): 17–43, 10.20517/CDR.2020.79. PMC8319838 34337348

[38] A. S. Nugraha , T. H. Y. Lam , H. Wongso , L. N. Firli , and P. A. Keller , “Lichens,” in Chemistry, Biology and Pharmacology of Lichen (Wiley, 2024), 193–229, 10.1002/9781394190706.CH14.

[39] A. H. Tripathi , N. Negi , R. Gahtori , et al., “A Review of Anti‐Cancer and Related Properties of Lichen‐Extracts and Metabolites,” Anti‐Cancer Agents in Medicinal Chemistry 22 (2022): 115–142, 10.2174/1871520621666210322094647. 34225637

[40] M. Mohammadi , V. Zambare , Z. Suntres , and L. Christopher , “Isolation, Characterization, and Breast Cancer Cytotoxic Activity of Gyrophoric Acid From the Lichen Umbilicaria Muhlenbergii,” PRO 10 (2022): 1361, 10.3390/PR10071361/S1.

[41] T. U. H. Dar , S. A. Dar , S. U. Islam , et al., “Lichens as a Repository of Bioactive Compounds: An Open Window for Green Therapy Against Diverse Cancers,” Seminars in Cancer Biology 86 (2022): 1120–1137, 10.1016/J.SEMCANCER.2021.05.028. 34052413

[42] B. Emsen , A. Aslan , B. Togar , and H. Turkez , “In Vitro Antitumor Activities of the Lichen Compounds Olivetoric, Physodic and Psoromic Acid in Rat Neuron and Glioblastoma Cells,” Pharmaceutical Biology 54 (2016): 1748–1762, 10.3109/13880209.2015.1126620. 26704132

[43] B. Emsen , A. Aslan , H. Turkez , A. Joughi , and A. Kaya , “The Anti‐Cancer Efficacies of Diffractaic, Lobaric, and Usnic Acid: In Vitro Inhibition of Glioma,” Journal of Cancer Research and Therapeutics 14 (2018): 941–951, 10.4103/0973-1482.177218. 30197329

[44] H. ‐J. Kim , S. ‐S. Suh , J. Park , et al., “7β‐22 Dihydroxyhopane, Isolated From the Sub‐Antarctic Lichen, Inhibits the Viability and Stemness in Glioma Stem Like Cells,” OncoTargets and Therapy 15 (2022): 1375–1383, 10.2147/ott.s371042. PMC9675426 36411942

[45] M. Varlı , M. Ji , E. Kim , et al., “Emodin Disrupts the KITENIN Oncogenic Complex by Binding ErbB4 and Suppresses Colorectal Cancer Progression in Dual Blockade With KSRP‐Binding Compound,” Phytomedicine 136 (2025): 156247, 10.1016/J.PHYMED.2024.156247. 39586126

[46] T. Bae , S. P. Hallis , and M. K. Kwak , “Hypoxia, Oxidative Stress, and the Interplay of HIFs and NRF2 Signaling in Cancer,” Experimental & Molecular Medicine 56, no. 3 (2024): 501–514, 10.1038/s12276-024-01180-8. PMC10985007 38424190

[47] O. Méndez , J. Zavadil , M. Esencay , et al., “Knock Down of HIF‐1α in Glioma Cells Reduces Migration In Vitro and Invasion In Vivo and Impairs Their Ability to Form Tumor Spheres,” Molecular Cancer 9 (2010): 133, 10.1186/1476-4598-9-133. PMC2896954 20515450

[48] S. Y. Lee , E. K. Jeong , M. K. Ju , et al., “Induction of Metastasis, Cancer Stem Cell Phenotype, and Oncogenic Metabolism in Cancer Cells by Ionizing Radiation,” Molecular Cancer 16, no. 1 (2017): 1–25, 10.1186/S12943-016-0577-4. PMC5282724 28137309

[49] N. H. Boyd , A. N. Tran , J. D. Bernstock , et al., “Glioma Stem Cells and Their Roles Within the Hypoxic Tumor Microenvironment,” Theranostics 11 (2021): 665–683, 10.7150/THNO.41692. PMC7738846 33391498

[50] Q. Zhang , Z. Han , Y. Zhu , J. Chen , and W. Li , “Role of Hypoxia Inducible Factor‐1 in Cancer Stem Cells (Review),” Molecular Medicine Reports 23 (2021): 1, 10.3892/MMR.2020.11655. PMC7673349 33179080

[51] S. Agnihotri and G. Zadeh , “Metabolic Reprogramming in Glioblastoma: The Influence of Cancer Metabolism on Epigenetics and Unanswered Questions,” Neuro‐Oncology 18 (2016): 160–172, 10.1093/NEUONC/NOV125. PMC4724176 26180081

[52] M. Varlı , S. J. Kim , M. G. Noh , et al., “KITENIN Promotes Aerobic Glycolysis Through PKM2 Induction by Upregulating the c‐Myc/hnRNPs Axis in Colorectal Cancer,” Cell & Bioscience 13 (2023): 1–21, 10.1186/S13578-023-01089-1/FIGURES/7. PMC10410973 37553596

[53] H. Yang , J. T. Zhong , S. H. Zhou , and H. M. Han , “Roles of GLUT‐1 and HK‐II Expression in the Biological Behavior of Head and Neck Cancer,” Oncotarget 10 (2019): 3066–3083, 10.18632/ONCOTARGET.24684. PMC6508962 31105886

[54] K. Kanwore , K. Kanwore , G. K. Adzika , et al., “Cancer Metabolism: The Role of Immune Cells Epigenetic Alteration in Tumorigenesis, Progression, and Metastasis of Glioma,” Frontiers in Immunology 13 (2022): 831636, 10.3389/FIMMU.2022.831636. PMC8980436 35392088

[55] P. Wang , L. Zhao , S. Gong , et al., “HIF1α/HIF2α–Sox2/Klf4 Promotes the Malignant Progression of Glioblastoma via the EGFR–PI3K/AKT Signaling Pathway With Positive Feedback Under Hypoxia,” Cell Death & Disease 12 (2021): 312, 10.1038/s41419-021-03598-8. PMC7990922 33762574

[56] Y. He , M. M. Sun , G. G. Zhang , et al., “Targeting PI3K/Akt Signal Transduction for Cancer Therapy,” Signal Transduction and Targeted Therapy 6, no. 1 (2021): 1–17, 10.1038/s41392-021-00828-5. PMC8677728 34916492

[57] B. Kaur , F. W. Khwaja , E. A. Severson , S. L. Matheny , D. J. Brat , and E. G. Van Meir , “Hypoxia and the Hypoxia‐Inducible‐Factor Pathway in Glioma Growth and Angiogenesis,” Neuro‐Oncology 7 (2005): 134–153, 10.1215/S1152851704001115. PMC1871894 15831232

[58] W. Zundel , C. Schindler , D. Haas‐Kogan , et al., “Loss of PTEN Facilitates HIF‐1‐Mediated Gene Expression,” Genes & Development 14 (2000): 391–396, 10.1101/gad.14.4.391. PMC316386 10691731

[59] D. D. Cheng , H. G. Zhao , Y. S. Yang , T. Hu , and Q. C. Yang , “GSK3β Negatively Regulates HIF1α mRNA Stability via Nucleolin in the MG63 Osteosarcoma Cell Line,” Biochemical and Biophysical Research Communications 443 (2014): 598–603, 10.1016/J.BBRC.2013.12.020. 24333432

[60] D. Flügel , A. Görlach , C. Michiels , and T. Kietzmann , “Glycogen Synthase Kinase 3 Phosphorylates Hypoxia‐Inducible Factor 1α and Mediates Its Destabilization in a VHL‐Independent Manner,” Molecular and Cellular Biology 27 (2007): 3253–3265, 10.1128/MCB.00015-07. PMC1899978 17325032

[61] I. Paw , R. C. Carpenter , K. Watabe , W. Debinski , and H. W. Lo , “Mechanisms Regulating Glioma Invasion,” Cancer Letters 362 (2015): 1–7, 10.1016/J.CANLET.2015.03.015. PMC4435977 25796440

[62] J. Zhang , F. G. Stevens , and D. T. Bradshaw , “Temozolomide: Mechanisms of Action, Repair and Resistance,” Current Molecular Pharmacology 5 (2012): 102–114, 10.2174/1874467211205010102. 22122467

[63] U. Herrlinger , T. Tzaridis , F. Mack , et al., “Lomustine‐Temozolomide Combination Therapy Versus Standard Temozolomide Therapy in Patients With Newly Diagnosed Glioblastoma With Methylated MGMT Promoter (CeTeG/NOA‐09): A Randomised, Open‐Label, Phase 3 Trial,” Lancet 393 (2019): 678–688, 10.1016/S0140-6736(18)31791-4. 30782343

[64] L. Shi , X. Fei , and Z. Wang , “Demethoxycurcumin Was Prior to Temozolomide on Inhibiting Proliferation and Induced Apoptosis of Glioblastoma Stem Cells,” Tumour Biology 36 (2015): 7107–7119, 10.1007/S13277-015-3427-X. 25877753

[65] C. Trejo‐Solís , N. Serrano‐Garcia , Á. Escamilla‐Ramírez , et al., “Autophagic and Apoptotic Pathways as Targets for Chemotherapy in Glioblastoma,” International Journal of Molecular Sciences 19 (2018): 3773, 10.3390/IJMS19123773. PMC6320836 30486451

[66] Q. Xia , M. Xu , P. Zhang , L. Liu , X. Meng , and L. Dong , “Therapeutic Potential of Autophagy in Glioblastoma Treatment With Phosphoinositide 3‐Kinase/Protein Kinase B/Mammalian Target of Rapamycin Signaling Pathway Inhibitors,” Frontiers in Oncology 10 (2020): 572904, 10.3389/FONC.2020.572904. PMC7567033 33123479

[67] V. M. Bedell , P. Dubey , H. B. Lee , et al., “Zebrafishology, Study Design Guidelines for Rigorous and Reproducible Data Using Zebrafish,” Communications Biology 8, no. 1 (2025): 1–14, 10.1038/s42003-025-07496-z. PMC12075475 40360750

[68] J. A. Geisler , J. M. Spehar , S. A. Steck , et al., “Modeling Brain Metastases Through Intracranial Injection and Magnetic Resonance Imaging,” Journal of Visualized Experiments 160 (2020): e61272, 10.3791/61272. PMC8787807 32568247

