# Supplementary material for: Methyl 4‐O‐Demethylbarbatate Destabilises HIF‐1α and Enhances Temozolomide Sensitivity in Glioma Cells
Source: Cell Prolif. 2026 Sep 27:e70279. Online ahead of print. doi: 10.1111/cpr.70279 (PMC13616830; doi:10.1111/cpr.70279)
Supplement: Supplementary file 1 [file CPR-9999-e70279-s001.docx]

**Methyl 4-*O*-demethylbarbatate Targets HIF-1α to Inhibit Glioma Cell Motility, Stemness, and Metabolism, and Synergistically Enhances the Effects of Temozolomide**

Rui Zhou ^1, #^, Mücahit Varlı ^1,2 #^, Rundong Liu ^3, 4^, Jieun Yu ^5^, Hyung-Ho Ha ^1^, Jae-Seoun Hur ^3^, Sang Kyum Kim ^5^, Hahyun Park ^1^, Hangun Kim ^1, *^

^1^College of Pharmacy, Sunchon National University, Sunchon, Jeollanam-do, Republic of Korea; zhourui274@gmail.com (R.Z.); mucahitvarli@s.scnu.ac.kr (M.V.); chuimi360@gmail.com (N.K.); hhha@sunchon.ac.kr (H.-H.H.); hhpark@sunchon.ac.kr (H.P.)

^2^Department of Microbiology, Tumor and Cell Biology, Karolinska Institutet, Stockholm, Sweden

^3^Korea Lichen Research Institute, Sunchon National University, Sunchon, Republic of Korea; lrdlichen@gmail.com (R.L.); jshur1@sunchon.ac.kr (J.-S.H.)

^4^Institute of Ocean Research, Ningbo Institute of Marine Medicine, Peking University, Ningbo, Zhejiang, China; lrdlichen@gmail.com (R.L.)

^5^College of Pharmacy, Chungnam National University, Daejeon, Republic of Korea; rhdwn0117@hanmail.net (J.Y.); sangkim@cnu.ac.kr (S.K.K.)

^#^Rui Zhou and Mücahit Varlı contributed equally to this work and share first authorship

^*^Correspondence: hangunkim@sunchon.ac.kr; Tel.: +82-61-750-3761 **LC-MS/MS analysis for drug distribution**

Stock solution (10 mg/mL) of Me-4-*O*-DBA was dissolved in DMSO. The standard working solutions were serially diluted to 0, 3.9, 15.6, 62.5, 250, and 1000 nM in 50% acetonitrile. All *in vivo* experiment were established using male C57BL/6 mouse. After 7 days of inoculation with GL261 cells, the mice were divided into control and experimental groups. The control group was injected with DMSO/PBS intraperitoneally. The experimental groups were injected with 5, 10 or 20 mg/kg of Me-4-*O*-DBA/PBS. Mice brains and plasma samples were collected after 24 h (n = 4 each). 100 mg of tissue samples were washed with cold-water and then collected in a 2 mL Falcon tube. Thereafter, three volumes of ice-cold deionized water were added, and the tissues were homogenized with the use of a vortexer bead beating by using an ultrasonic homogenizer (Bioprep-24 R, ALLSHENG, China) at 4°C for 5 min. 300 μL of blood was collected from the orbital by using a heparinized capillary tube into the corner of the eye socket underneath the eyeball. All the homogenates were centrifuged at 13000 rpm for 5 min. Supernatants were separated from tissues pellet and kept for LC-MS/MS analysis.

**Supplementary Figure S1.** ^1^H NMR spectrum of methyl 4-*O*-demethylbarbatate (Me-4-*O*-DBA) in DMSO.

**Supplementary Figure S2.** ^1^H NMR spectrum of methyl 4-*O*-demethylbarbatate (Me-4-*O*-DBA) in DMSO, D_2_O.

**Supplementary Figure S3.** ESIMS spectrum of Me-4-O-DBA.

**Supplementary Figure S4.** Distribution of Me‑4‑O‑DBA following intraperitoneal injections at doses of 5, 10, or 20 mg/kg every other day for 2 weeks. Quantitative LC-MS/MS analysis of Me‑4‑O‑DBA levels in the plasma **(A)** and brain **(B)** of C57BL/6 mice.

**Table S1.** Primer sequences for qRT-PCR.

| **Gene**  **symbol** | **Primers sequences** | |
| --- | --- | --- |
|  | **For (5'-3')** | **Rev (5'-3')** |
| *GLUT1* | CTTTGTGGCCTTCTTTGAAGT | CCACACAGTTGCTCCACAT |
| *HK2* | AAGGCTTCAAGGCATCTG | CCACAGGTCATCATAGTTCC |
| *PKM2* | ATTATTTGAGGAACTCCGCCGCCT | ATTCCGGGTCACAGCAATGATGG |
| *LDHA* | TGGCCTGTGCCATCAGTATC | TTCCAAGCCACGTAGGTCAA |
| *HIF-1α* | ATCCATCTGACCATGAGGAAATG | TCGGCTAGTTAGGGTACACTTC |
| *c-Myc* | AATGAAAAGGCCCCCAAGGTAGTTATCC | GTCGTTTCCGCAACAAGTCCTCTTC |
| *CD44* | TGCCGCTTTGCAGGTGTAT | GGCCTCCGTCCGAGAGA |
| *β-actin* | ATTGTGAACTTTGGGGGATG | GATGAGATTGGCATGGCTTT |
| *N-cadherin* | CTCCTATGAGTGGAACAGGAACG | TTGGATCAATGTCATAATCAAGTGCTGTA |
| *SNAIL* | TCCCGGGCAATTTAACAATG | TGGGAGACACATCGGTCAGA |
| *TWIST* | CGGGAGTCCGCA GTCTTA | TGAATCTTGCTCAGCTTGTC |
| *MSI1* | ACCAAGAGATCCAGGGGTTT | TCGTTCGAGTCACCATCTTG |
| *ALDH1* | TGTTAGCTCATGCCGACTTG | TTCTTAGCCCGCTCAACACT |
| *NESTIN* | GGCGCACCTCAAGATGTCC | CTTGGGGTCCTGAAAGCTG |
| *OCT4* | GACCATCTGCCGCTTTGAGGCTCTG | GCGCCG GTTACAGAACCACACTCG |
| *CHD4* | CACCGAATCCTCAACCA | GGCTGTCGCTCATACTT |
| *SRC* | CTCATGGTGTGGCTCGTTCATC | GCTCTGCTGGCGGTTTATTCTG |
| *PGC-1α* | GTGTCGCCTTCTTGTTCTTCTTTT | CGCATCCTTTGGGGTCTTT |
| *PDK1* | CATGTCACACGTGGGTAATGAGG | CTCAACACGAGGTCTTGGTGA |

**Table S2.** Antibody information.

| **Antibody name** | **Product Information** |
| --- | --- |
| Anti-Twist antibody | Abcam, ab49254 |
| Anti-snail+slug antibody | Abcam, ab180714 |
| Beta-catenin antibody | Cell signaling, #9562 |
| Alpha tubulin (11h101) Rabbit mAb | Cell signaling, #2125 |
| Goat Anti-Rabbit IgG (H+L) Peroxidase Conjugated | Thermo scientific, 31460 |
| Goat Anti-Mouse IgG (H+L) Peroxidase Conjugated | Thermo scientific, NCI1430KR |
| Phospho-STAT3 (Try705) (D3A7) XP Rabbit Antibody | Cell signaling, #9145 |
| STAT3 (D3Z2G) Rabbit mAB | Cell signaling, #12640 |
| PKM2 (D78A4) XP Rabbit mAB | Cell signaling, #4053 |
| GLUT1 (D3J3A) Rabbit mAB | Cell signaling, #12939 |
| Phospho-AKT (Ser473) (D9E) XP Rabbit Antibody | Cell signaling, #4060 |
| Phospho-mTOR (Ser2448) (D9C2) XP Rabbit Antibody | Cell signaling, #5536 |
| MTOR (7C10) Rabbit mAB | Cell signaling, #2983 |
| HIF1A (D1S7W) XP Rabbit mAB | Cell signaling, #36169 |
| LDHA (C4B5) Rabbit mAB | Cell signaling, #3582 |
| Beta-actin antibody | Cell signaling, #4967 |
| Akt (pan) (C67E7) Rabbit mAb | Cell signaling, #4691 |
| Purified mouse anti-N-cadherin | BD Biosiences, 610921 |
| Hexokinase II (C64G5) Rabbit mAb | Cell signaling, #2867 |
| SAPK/JNK Antibody | Cell signaling, #9252 |
| Phospho-SAPK/JNK (Thr183/Tyr185) (81E11) Rabbit mAb | Cell signaling, #4668 |
| Anti-cyclin D1 Ab-3 mouse mAB (DCS-6) | Merck, Kenilworth, NJ, USA |
| ALDH1/2 (H8) | sc166362; Santa Cruz Biotechnology, Dallas, TX, USA |
| AntiGPCR GPR49 antibody (EPR3065Y), Lgr-5 | ab75850; Abcam, Cambridge, MA, USA |
| Anti-Musashi 1 / Msi1 (EP1302 | ab52865, Abcam |
| Gli1 (H-300) | sc-20687; SANTA CRUZ, Dallas, TX, USA |
| Gli2 (C-10) | sc-271786; SANTA CRUZ |
| Bcl-xL Antibody | Cell signaling, #2762 |
| PARP Antibody | Cell signaling, #9542 |
| Caspase-3 Antibody | Cell signaling, #9662 |
| Phospho-EGF Receptor (Tyr1068) Antibody | Cell signaling, #2234 |
| c-Jun (60A8) Rabbit mAb | Cell signaling, #9165 |
| c-Fos Antibody | Cell signaling, #4384 |
| PD-L1 (E1L3N) XP Rabbit mAb | Cell signaling, #13684 |
| SMAD4 |  |
| HSP70 (6B3) Rat mAB | Cell signaling, #4873 |
| HSP90 (C45G5) Rabbit mAB | Cell signaling, #4877 |
| Goat Anti-Rat IgG (H+L) Peroxidase Conjugated | Thermo scientific, 31470 |
| CD44 (156-3C11) Mouse mAb | Cell signaling, #3570 |
| Anti-SNAIL + SLUG antibody | ab180714 |
| Anti-alpha smooth muscle Actin antibody | ab5694 |
| Ubiquitin (P4D1) | Santa Cruz Biotechnology, sc-8017 |
